# Blau syndrome NOD2 mutations result in loss of NOD2 cross-regulatory function

**DOI:** 10.3389/fimmu.2022.988862

**Published:** 2022-09-15

**Authors:** Liming Mao, Atika Dhar, Guangxun Meng, Ivan Fuss, Kim Montgomery-Recht, Zhiqiong Yang, Qiuyun Xu, Atsushi Kitani, Warren Strober

**Affiliations:** ^1^ Mucosal Immunity Section, Laboratory of Clinical Immunology and Microbiology, National Institute of Allergy and Infectious Diseases, National Institutes of Health, Bethesda, MD, United States; ^2^ Department of Immunology, School of Medicine, Nantong University, Nantong, China; ^3^ The Center for Microbes, Development and Health, Chinese Academy of Sciences (CAS) Key Laboratory of Molecular Virology and Immunology, Institut Pasteur of Shanghai, University of Chinese Academy of Sciences, Shanghai, China; ^4^ Pasteurien College, Soochow University, Suzhou, China; ^5^ Clinical Research Directorate/Clinical Monitoring Research Program, Leidos Biomedical Research, Inc., National Cancer Institute (NCI) Campus at Frederick, Frederick, MD, United States

**Keywords:** Blau, Nod2, IRF4, NFkapapB, Crohns disease

## Abstract

The studies described here provide an analysis of the pathogenesis of Blau syndrome and thereby the function of NOD2 as seen through the lens of its dysfunction resulting from Blau-associated NOD2 mutations in its nucleotide-binding domain (NBD). As such, this analysis also sheds light on the role of NOD2 risk polymorphisms in the LRR domain occurring in Crohn’s disease. The main finding was that Blau NOD2 mutations precipitate a loss of canonical NOD2 signaling *via* RIPK2 and that this loss has two consequences: first, it results in defective NOD2 ligand (MDP)-mediated NF-κB activation and second, it disrupts NOD2-mediated cross-regulation whereby NOD2 downregulates concomitant innate (TLR) responses. Strong evidence is also presented favoring the view that NOD2-mediated cross-regulation is under mechanistic control by IRF4 and that failure to up-regulate this factor because of faulty NOD2 signaling is the proximal cause of defective cross-regulation and the latter’s effect on Blau syndrome inflammation. Overall, these studies highlight the role of NOD2 as a regulatory factor and thus provide additional insight into its function in inflammatory disease. Mutations in the nucleotide binding domain of the *CARD15* (NOD2) gene underlie the granulomatous inflammation characterizing Blau syndrome (BS). In studies probing the mechanism of this inflammation we show here that NOD2 plasmids expressing various Blau mutations in HEK293 cells result in reduced NOD2 activation of RIPK2 and correspondingly reduced NOD2 activation of NF-κB. These *in vitro* studies of NOD2 signaling were accompanied by *in vivo* studies showing that BS-NOD2 also exhibit defects in cross-regulation of innate responses underlying inflammation. Thus, whereas over-expressed intact NOD2 suppresses TNBS-colitis, over-expressed BS-NOD2 does not; in addition, whereas administration of NOD2 ligand (muramyl dipeptide, MDP) suppresses DSS-colitis in Wild Type (WT) mice it fails to do so in homozygous or heterozygous mice bearing a NOD2 Blau mutation. Similarly, mice bearing a Blau mutation exhibit enhanced anti-collagen antibody-induced arthritis. The basis of such cross-regulatory failure was revealed in studies showing that MDP-stimulated cells bearing BS-NOD2 exhibit a reduced capacity to signal *via* RIPK2 as well as a reduced capacity to up-regulate IRF4, a factor shown previously to mediate NOD2 suppression of NF-κB activation. Indeed, TLR-stimulated cells bearing a Blau mutation exhibited enhanced *in vitro* cytokine responses that are quieted by lentivirus transduction of IRF4. In addition, enhanced anti-collagen-induced joint inflammation in mice bearing a Blau mutation was accompanied by reduced IRF4 expression in inflamed joint tissue and IRF4 expression was reduced in MDP-stimulated cells from BS patients. Thus, inflammation characterizing Blau syndrome are caused, at least in part, by faulty canonical signaling and reduce IRF4-mediated cross-regulation.

## Introduction

Blau Syndrome (BS) is an autosomal dominant disorder caused by mutations in *Card15*, the gene encoding the NOD-like Receptor (NLR) protein, NOD2, and is characterized by granulomatous inflammation of the skin, joints and eyes, but rarely, if ever, the GI tract (1, 2). This clinical pattern differs very considerably from that characterizing Crohn’s disease (CD), a GI-centered disease, despite the fact that a subgroup of Crohn’s disease (CD) patients also bear *Card15* abnormalities (in the form of polymorphisms). This difference is most likely due to the fact that the *Card15* genetic abnormalities occurring in the two diseases differ with respect to the *Card15* domain exhibiting mutational hits: the CD *Card15* genetic abnormalities are present in the LRR domain of *Card15*, the domain involved in NOD2 ligand engagement, whereas BS *Card15* genetic abnormalities are present in the nucleotide-binding domain (NBD) of *Card15*, the domain mediating NOD2 oligomerization and interaction with downstream adaptors (3). These differing genetic abnormalities have led to the view that the mutations (polymorphisms) in CD cause a loss-of-function abnormality due to an impairment in the ability of NOD2 to recognize its ligand, muramyl dipeptide (MDP), whereas the mutations in BS cause a gain-of-function abnormality due to NOD2 hyper/auto-activation in the absence or presence of MDP. In the case of CD this view is supported by functional studies (see further discussion below) but in the case of BS the functional data are cloudy in that it is not clear that BS NBD mutations are a direct cause of excessive NOD2 activation and down-stream signaling. Whereas the latter is supported by *in vitro* studies showing that plasmids expressing Blau constructs mediate increased NF-κB activation independent of NOD2 ligand stimulation (4–6) and molecular studies suggesting that NBD mutations lead to molecular instability (7), it is not supported by reports that cells from Blau patients mount poor responses when cultured with MDP (8–10).

Current understanding of the mechanisms by which *CARD15* polymorphisms increase the risk of developing Crohn’s disease provides some insight into how Blau mutations might lead to multi-organ inflammation. The most accepted of such mechanisms holds that *CARD15* abnormalities cause reduced NOD2 function that affects NOD2 responses elicited by MDP present in the cell walls of intestinal commensal organisms (11). These impaired innate immune responses then render the individual bearing the CD abnormality less able to prevent commensal organisms from colonizing the lamina propria and inducing immune responses that cause mucosal inflammation at this site. Along the same lines, there is evidence that this unresponsiveness leads to intestinal dysbiosis and thus a microflora enriched in commensal organisms that cause inflammation and/or depleted of commensal organisms that prevent inflammation (12). Another mechanism by which NOD2 dysfunction caused by *CARD15* polymorphisms increase risk of Crohn’s disease

is based on studies showing that NOD2 signaling results in cross-regulation (i.e., down-regulation) of TLR or other innate responses in the intestinal milieu and prevents excessive TLR stimulation at this site (13, 14); thus, disruption of this regulatory function due to NOD2 silencing causes inflammation. Evidence in favor of such NOD2 regulatory function came initially from the fact that while MDP enhances concomitant TLR responses it inhibits these TLR responses when present prior to the TLR stimulus; in addition, both over-expression of NOD2 or administration of MDP (i.e., procedures that enhance NOD2 signaling) inhibit experimental colitis (15). Finally, recent studies have provided evidence that NOD2 can, in fact, mediate inhibitory function by upregulating IRF4, a factor that inhibits NF-κB activation by affecting polyubiquitination of NF-κB adaptors (16). This second mechanism of NOD2 dysfunction, wherein NOD2 fails to exert down-regulation of innate responses may be more relevant to Blau Syndrome since the latter is an inflammatory disease occurring in non-mucosal, sterile tissue sites and is therefore less likely to be influenced by a bacterial microflora.

In the present study we explored the relation of Blau-type *CARD15* mutations to inflammatory disease with studies of NOD2 signaling in cells transfected with plasmids expressing various Blau mutations and established that these mutations had adverse effects on NOD2-induced phosphorylation and/or ubiquitination of RIPK2, the downstream signaling intermediate of NOD2; as a result, these mutations had a negative effect on MDP-induced activation of NF-κB and MAPK. We then showed that this signaling defect is accompanied by impaired *in vivo* NOD2 cross-regulation of innate responses which was traceable to decreased upregulation of IRF4, the above-mentioned regulatory factor. These data therefore suggest that the multi-faceted inflammation characterizing Blau syndrome is attributable to loss of NOD2-mediated immune regulation.

## Results

### NOD2 mutations occurring in Blau syndrome (BS-NOD2 mutations) cause defects in NOD2 oligomerization

As well described by Jun et al. (17), the NOD2 signaling pathway initiated by interaction of its LRR domain with MDP is followed by NOD2 oligomerization and binding to RIPK2 (RICK), its immediate downstream signaling partner (18, 19). This, in turn, is followed by RIPK2 activation marked by RIPK2 tyrosine phosphorylation and K63-linked polyubiquitination as well as by subsequent RIPK2 interaction with TRAF6 and other signaling components of NF-κB (20). In addition, it is followed by RIPK2 interaction with IRF4, a component involved in NOD2 regulatory activity (16).

**Figure 1:**
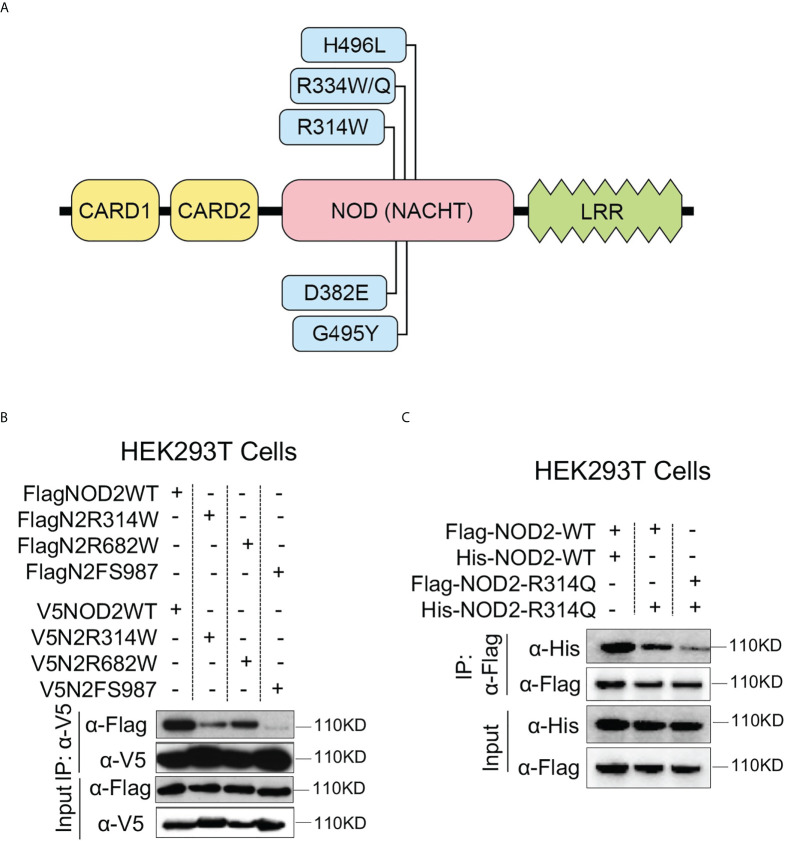
The BS-NOD2 Mutation Causes Reduced NOD2 Self-Interaction. **(A)** Diagram of NOD2 structure showing main NOD2 domains and locations of nucleotide binding domain Blau mutations in the Blau constructs studied here. **(B)** HEK293T cells were transfected with constructs expressing Flag- or V5-tagged mouse wildtype NOD2 or Blau syndrome-associated mutations (FlagN2R314W, FlagN2R682W) or NOD2 with frameshift mutation (FlagN2FS987) with different combinations. The cells were lysed 24 hours post transfection and the cell lysates were subjected to IP using an anti-V5 antibody and Western blotting for NOD2 detection. **(C)** HEK293T cells were transfected with constructs expressing flag- or his-tagged mouse wild type NOD2 or NOD2 with a R314Q mutation with different combinations. The cells were lysed 24 hours post transfection and the cell lysates were subjected to IP using an anti-flag antibody and Western blotting for NOD2 detection.

On the basis of this knowledge of NOD2 signaling, we first determined the impact of a Blau mutation or a CD-frameshift mutation on the ability of the mutated NOD2 to interact with itself (i.e., to oligomerize) or to interact with wild type (WT) NOD2. To this end, we co-transfected vectors expressing Flag-tagged or V5-tagged WT NOD2 and similarly tagged mutated NOD2 into HEK293T cells and then performed immunoprecipitation/immunoblotting assays to assess binding of the Flag-tagged NOD2 with the V5-tagged NOD2. A diagram showing the major domains of the NOD2 gene as well as the locations of the mutations in the constructs employed in these studies and in subsequent studies is provided in **Figure 1A**.

We found that co-expression of Flag-tagged NOD2 and V5-tagged NOD2 each bearing a Blau mutation (R314W) gave a diminished but not absent signal compared to co-expression of differentially marked WT NOD2 indicating decreased ability of NOD2 with a Blau mutation to bind with itself (**Figure 1B**). There was also a decreased but not absent ability of NOD2 with the same Blau mutation to bind to WT NOD2 (data not shown). Using the same differential tagging strategy, NOD2 with both R682W and FS987 LRR mutations also showed less interaction with itself than WT NOD2 (especially the FS987 mutation) (**Figure 1B**). In a second and similar co-transfection study in which tagged vectors expressing NOD2 bearing a R314Q mutation were transfected into HEK293T cells we found that co-expression of His-tagged Blau NOD2 (R314Q) and Flag-tagged WT NOD2 gave a diminished signal compared to co-expression of Flag-tagged WT NOD2 and His-tagged WT NOD2 (**Figure 1C**); in addition, co-transfection of Flag-tagged Blau NOD2 (R314Q) with His-tagged Blau NOD2 (R314Q) gave an even more diminished signal (**Figure 1C**). These results cannot be explained by increased degradation of Blau constructs as none were detected diminution of the input signal or by Western blot (not shown). Thus, they provide evidence that Blau mutations diminish but do not completely inhibit the initial step of NOD2 signaling, namely NOD2 self-oligomerization.

### The BS-NOD2 mutation causes reduced NOD2 interaction with RIPK2 and reduced RIPK2 tyrosine phosphorylation and/or K63 polyubiquitination

**Figure 2:**
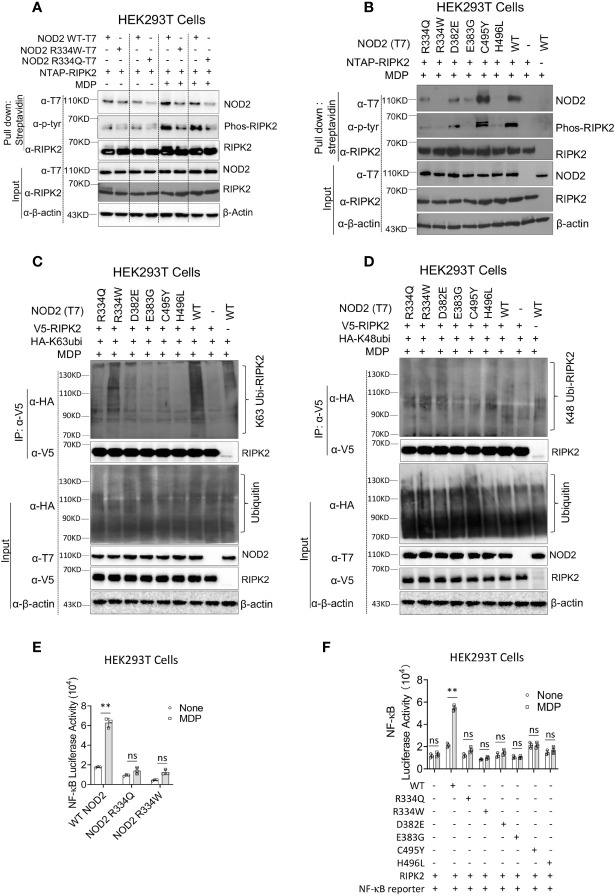
The BS-NOD2 Mutation Causes Reduced NOD2-Induced Interaction with RIPK2, Reduced RIPK2 Activation and Reduced NF-κB Activation. **(A)** HEK293T cells were transfected with constructs expressing T7-tagged wildtype or BS-NOD2 mutations (R334Q or R334W) and NTAP-tagged RIPK2; 24 hours later, the cells were incubated with or without MDP (10μg/ml) for 6 hours after which the cells were lysed and subjected to pull-down assay for detecting NOD2-RIPK2 interaction and RIPK2 phosphorylation. **(B)** HEK293T cells were transfected with constructs expressing T7-tagged human wildtype NOD2 or with one of multiple constructs expressing various NOD2 mutations associated with Blau syndrome; in addition, the cells were transfected with NTAP-tagged RIPK2; 24 hours later, the cells were stimulated with MDP (10μg/ml) for 6 hours after which the cells were lysed and the cell lysates obtained were subjected to pull-down assay using streptavidin beads and Western blotting for detection of NOD2-RIPK2 interaction and RIPK2 phosphorylation. **(C, D)** HEK293T cells were transfected with constructs expressing wildtype NOD2 or one of multiple Blau syndrome constructs expressing various NOD2 mutations; in addition, the cells were transfected with constructs expressing V5-tagged RIPK2 and HA- tagged K63-only ubiquitin (**C**, other lysine sites mutated to alanine) or HA-tagged K48-only ubiquitin (**D**, other lysine sites mutated to alanine); 24 hours later, the cells were stimulated with MDP (10μg/ml) for 6 hours and then lysed; finally, the cell lysates were subjected to IP with agarose beads conjugated with anti-V5 antibody and Western blotting for detection of RIPK2 polyubiquitination. All data are representative figures from two independent experiments. **(E)** HEK293T cells were transfected with constructs expressing wildtype NOD2 or constructs bearing the indicated Blau mutations as well as an NF-κB reporter construct; 24 hours later, the cells were incubated with or without MDP (10μg/ml) for 6 hours and were then lysed; finally, the lysates were subjected to luciferase assay for detection of NF-κB activation. **(F)** HEK293T cells were transfected with constructs expressing one of multiple constructs expressing Blau syndrome NOD2 mutations, RIPK2 and NF-κB reporter; 24 hours later, the cells were incubated with or without MDP (10μg/ml) for 6 hours and were then lysed; finally, the lysates were subjected to luciferase assay for detection of NF-κB activation. Studies in **(A, B)** were conducted with three biological replicates for each sample. Data were analyzed using a two-way ANOVA with Tukey’s multiple comparisons and are displayed as mean ± SEM; **p<0.01. ns, not significant.

As noted above, ligand-induced NOD2 oligomerization is followed by NOD2 interaction with RIPK2 and RIPK2 activation, the latter accompanied by RIPK2 phosphorylation and K63-polyubiquitination. To investigate if the BS-NOD2 mutation affects its interaction with and activation of RIPK2, an NTAP-RIPK2 construct together with constructs carrying wild type NOD2-T7 or BS-NOD2-T7 (R334W or R334Q) were co-transfected into HEK293T cells; 24 hours later, the cells were cultured with or without MDP for 6 hours and then lysed; finally, the cell lysates were subjected to pull-down using streptavidin beads followed by Western blotting with anti-NOD2-T7. The results showed that MDP stimulation of cells bearing WT NOD2 enhanced the interaction between NOD2 and RIPK2, whereas, in contrast, MDP stimulation of cells bearing NOD2 plasmid expressing the two different R334 BS mutations (R334W and R334Q) exhibited greatly reduced interactions with RIPK2 (**Figure 2A**, first row). These results were generalized by studies in which transfection of constructs with BS NOD2 mutations at multiple sites; here again BS-NOD2 mutations led to reduced NOD2 interactions with RIPK2 following MDP stimulation of transfected cells, except in the case of the C495Y mutation which was associated with increased interaction with RIPK2 (**Figure 2B**, first row).

In the same studies in which NOD2-RIPK2 interactions were assessed (as described above), cell lysates subjected to pull-down with streptavidin beads were also assessed by Western blotting with anti-phosphotyrosine to detect phosphorylated RIPK2. We found that in parallel with the binding studies described above, cells transfected with WT-NOD2 plasmid exhibited greatly enhanced RIPK2 phosphorylation following MDP stimulation, and that phosphorylation was greatly reduced in cells transfected with plasmids with the two mutations at the R334 site (**Figure 2A**, second row). Similarly, transfection of plasmids with BS NOD2 mutations at multiple sites also exhibited greatly reduced RIPK2 phosphorylation, again except in the case of the C495Y mutation which was associated with increased RIPK2 phosphorylation (**Figure 2B**, second row).

The above determination of the effect of BS mutations on RIPK2 phosphorylation was accompanied by studies assessing the effect of mutations on RIPK2 polyubiquitination. In this case constructs expressing RIPK2, WT-NOD2, BS-NOD2 expressing various mutations, and K63 or K48 ubiquitin were transfected into HEK293T cells; 24 hours later the cells were cultured with MDP for 6 hours and then lysed; finally, the cell lysates were subjected to immunoprecipitation followed by Western blotting. Cells transfected with plasmids expressing a variety of BS-NOD2 mutations, exhibited greatly reduced K63 polyubiquitination compared to cells expressing WT-NOD2 (**Figure 2C**); in contrast, RIPK2 K48-ubiquitination was not affected by these various mutations of NOD2 (**Figure 2D**). Interestingly, the BS-NOD2 mutation at C495 (C495Y) that alone among the various mutations examined exhibited enhanced NOD2-RIPK2 interaction and enhanced RIPK2 tyrosine phosphorylation following MDP stimulation (**Figures 2A, B**), exhibited little or no RIPK2 K63 polyubiquitination (**Figure 2C**). Thus, all Blau-associated NOD2 mutations exhibited impaired NOD2-RIPK2 interaction, as well as RIPK2 tyrosine phosphorylation and/or K63- polyubiquitination.

### The BS-NOD2 mutation causes reduced NOD2-induction of NF-κB activation

It seemed likely that the above negative effects of BS mutations on NOD2 oligomerization, binding to RIPK2 and RIPK2 phosphorylation and/or K63-polyubiquitination would also have a negative effect on NOD2 induction of NF-κB activation. To explore this possibility, HEK293T cells were transfected with plasmids expressing an NF-κB luciferase reporter, RIPK2 and plasmids expressing either WT-NOD2 or BS-NOD2 bearing the already well-studied Blau mutations (R334Q or R334W) and, 24 hours later, the cells were cultured with or without MDP for 6 hours; finally, the cells were lysed, and the lysates obtained were subjected to NF-κB luciferase assays. We found that MDP induced increased activation of NF-κB in cells transfected with wild type NOD2 but not in cells transfected with BS-NOD2 constructs; in addition, cells transfected with BS-NOD2 plasmids did not exhibit elevated baseline levels of NF-κB activation (**Figure 2E**). Similarly, cells transfected with plasmids expressing the same array of BS mutations as in the above phosphorylation and ubiquitination studies were also unresponsive to MDP with respect to NF-κB activation and exhibited no increase in baseline NF-κB activation (**Figure 2F**).

Overall, these *in vitro* studies of constructs expressing BS mutations uniformly exhibited virtually absent NOD2-mediated NF-κB activation in the presence of MDP stimulation and no increase in baseline NF-κB activation in the absence of MDP-stimulation. The validity of these findings is supported by the fact that in the HEK293 T cell transfection studies used to study NOD2 induction of NF-κB activation the signal produced in cells expressing NOD2 with Blau mutations did not exceed that in cells expressing exogenous RIPK2 (in the absence of either WT or Blau NOD2 expression. In addition, they are supported by physiologic *ex vivo* studies discussed below showing that cells from NOD2 KO mice bearing a transgene with a Blau mutation (R314W) exhibit greatly reduced MDP-induced NF-κB and MAPK responses (See **Supplementary Figures 1**, **2** ).

### Mice with transient over-expression of NOD2 bearing a Blau syndrome (BS)-associated mutation are not protected from induction of TNBS-colitis

We have previously shown that over-expression of wild type (WT) (intact) NOD2 but not NOD2 with a CD-frameshift LRR-domain polymorphism protects mice from the development of TNBS-colitis, presumably because intact NOD2 but not NOD2 with a polymorphism that interferes with ligand recognition provides NOD2 signaling that cross-regulates (i.e., downregulates) TLR responses and thus ameliorates the colitis (20, 21). Such over-expression studies thus proved to be a powerful approach to the assessment of NOD2 regulatory function.

**Figure 3:**
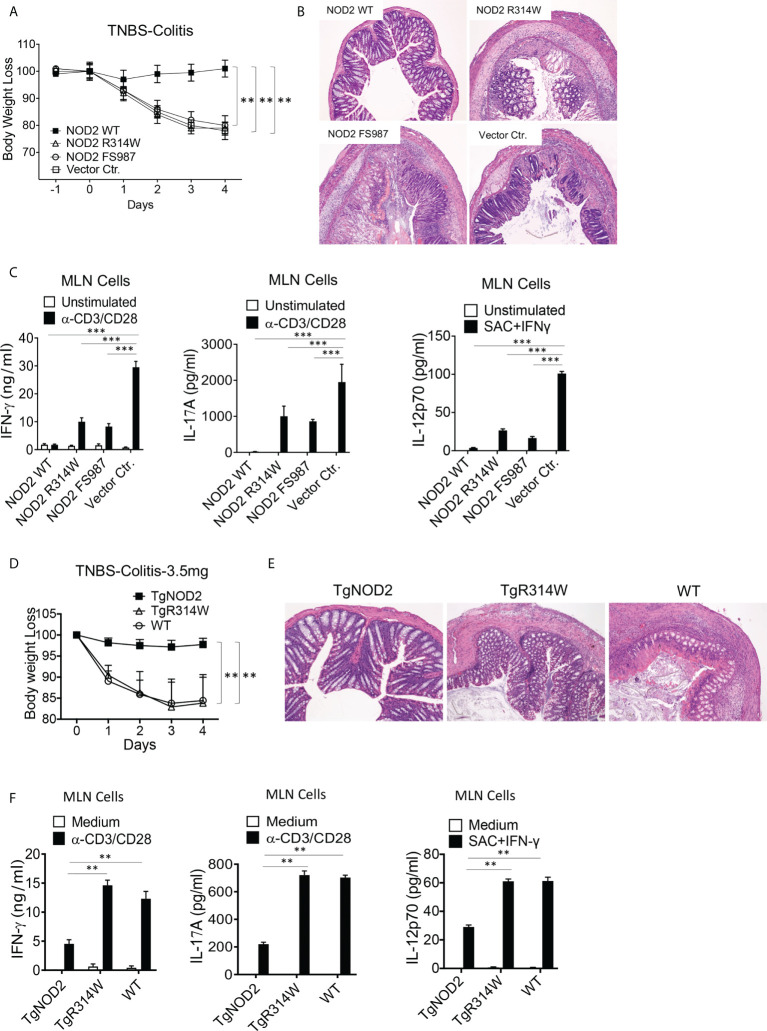
Mice with transient over-expression of Blau Syndrome (BS)-associated NOD2 mutation (R314W) or BS-NOD2 transgene are not protected from induction of TNBS-colitis. **(A)** TNBS-colitis associated weight loss of C57BL/10 mice administered TNBS per rectum (3.5 mg/mouse) on day 0 of study and plasmids expressing intact NOD2 (NOD2-WT), Blau syndrome-associated mutation of NOD2 (NOD2-R314W) or Crohn’s disease-associated NOD2 frameshift mutation (NOD2-FS98) encapsulated in HVJ-E or the empty vector control (Vector Ctr) on day -1, day 0 and day 1 of study; mice were harvested for analysis on day 4. **(B)** Representative histology of colons after H&E staining of tissues from mice harvested on day 4. **(C)** TCR-induced production of IFN-γ, IL-17A and SAC+IFN-γ-induced IL-12p70 production by extracted MLN cells from mice harvested on day 4 determined by ELISA. **(D)** TNBS-colitis associated weight loss of C57BL/10 mice bearing transgenes expressing intact NOD2 (TgNOD2, n=6) or NOD2-R314W (TgBlau, n=7) and wildtype (WT, n=9) mice administered TNBS per rectum (3.5 mg/mouse); mice were harvested for analysis on day 4. **(E)** Representative histology of colons after H&E staining of colonic tissue from mice harvested on day 4. **(F)** TCR-induced production of IFN-γ and IL-17A by extracted MLN cells harvested on day 4. Data in **(A, D)** were analyzed using a two-way ANOVA with Dunnett’s multiple comparisons. Data in **(C, F)** were analyzed using a two-way ANOVA with Tukey’s multiple comparisons. Data are displayed as mean ± SEM; **p<0.01, ***p<0.001.

To determine if NOD2 with a Blau mutation retains the cross-regulatory function of WT NOD2, we first conducted studies of TNBS-colitis in mice with transient over-expression of WT NOD2, Blau syndrome (BS)-NOD2 (bearing the NBD mutation: R314W), and CD-frameshift NOD2 (FS987). Accordingly, we administered TNBS intrarectally to C57BL/10 mice (3.5 mg/mouse) that were subjected to IP injections of plasmids expressing cDNA encoding the above forms of NOD2 encapsulated in HVJ-E, a viral envelop preparation that enables high level delivery of plasmid into hematopoietic cells (15, 22). Using body weight loss as a criterion of inflammation, we found that mice administered a WT NOD2 vector were protected from the development of TNBS-colitis whereas mice administered a CD-frameshift NOD2 (FS987), or empty vector were not protected (**Figure 3A**). In addition, by the same criterion, mice administered a BS-NOD2 (R314W) vector were also not protected (**Figure 3A**). These body weight data were corroborated by macro- and microscopic studies of colons at 4 days after TNBS administration in that colons of mice expressing WT-NOD2 manifested no evidence of inflammation whereas mice expressing a CD-frameshift or BS-NOD2 exhibited severe gastrointestinal inflammation characterized by intestinal shortening, massive lymphocyte infiltration of the lamina propria, increased peritoneal cell accumulation and reduced survival, i.e., changes equivalent to those occurring in mice administered a control vector (empty plasmid) (**Figure 3B** and **Supplementary Figures 1A–C**). In addition, anti-CD3/CD28-stimulated cells from mesenteric lymph nodes (MLN) of mice administered CD-frameshift- or BS-NOD2- vectors produced increased amounts of pro-inflammatory cytokines compared to cells from mice administered a WT NOD2 vector (**Figure 3C**). Finally, it should be noted that these findings were not due to different levels of over-expression of NOD2 since quantitative RT-PCR analysis of cells from each group of mice showed that mRNA expression levels of the various types of NOD2 were similar (**Supplementary Figure 1D**). Taken together, whereas WT NOD2 HVJ-E-mediated over-expression protected mice from induction of TNBS-colitis, similar BS-associated NOD2 over-expression did not; in this respect BS-NOD2 was similar to CD-frameshift NOD2.

### Mice expressing a transgene of NOD2 bearing a Blau-associated (R314W) mutation are not protected from TNBS-colitis

Complementary studies of the ability of over-expressed NOD2 to protect mice from TNBS-colitis were then conducted in transgenic mice expressing WT-NOD2 or BS-NOD2 transgenes containing a R314W mutation. To this end, NOD2 transgenes under an MHC-Class II promoter were introduced into C57BL/6 mice, after which the latter were back-crossed with C57BL/10 mice (for at least 6 generations) to obtain transgenic mice with a genetic background that supports robust TNBS-colitis. Sequencing of DNA amplified by PCR primers designed to selectively detect either genomic intact or mutant genes prior to their transcription in genomic DNA or both types of genes post-transcription in cDNA derived from total extracted RNA indicated that BS-NOD2 containing the R314W mutation was integrated into the transgenic mouse genome (**Supplementary Figures 1E, F**); in addition, NOD2-specific RT-PCR analysis showed that NOD2 expression levels of WT-NOD2 and BS-NOD2 were comparable in the respective transgenic mice and both were higher than NOD2 expression levels in WT mice (**Supplementary Figure 1G**).

In these studies of mice bearing transgenes, we found that whereas TNBS-colitis in C57BL/10 mice induced by intra-rectal injection of TNBS (3.5 mg/mouse) bearing a WT-NOD2 transgene were protected from induction of TNBS-colitis and exhibited virtually no colitis, mice bearing the BS-NOD2 transgene developed severe colitis similar in intensity to that induced in WT mice not bearing any transgene, as reflected by weight loss and microscopic evidence of colitis (**Figures 3D, E**). In addition, stimulated MLN cells from mice bearing a WT-NOD2 transgene exhibited reduced pro-inflammatory cytokine production as compared to MLN cells from mice bearing the BS-NOD2 transgene or WT mice not bearing a transgene (**Figure 3F**). Similar results were observed in C57BL/6 mice bearing the same transgenes, although, as expected in C57BL/6 mice, the TNBS-colitis induced by intra-rectal administration of 3.5 mg/mouse TNBS in WT mice was milder than that observed in the C57BL/10 mice administered this dose of TNBS (**Supplementary Figures 1H, I**). Thus, studies of transgenic mice carrying a transgene with BS-associated NOD2 mutation also support the view that over-expressed BS-NOD2 fails to protect mice from developing colitis upon TNBS challenge.

As mentioned above, previous studies involving over-expression of NOD2 in cell lines have provided data suggesting that NOD2 bearing a Blau mutation exhibits constitutive (MDP-independent) hyper-responsiveness (4–6). Thus, one possible explanation of the studies presented above (showing that over-expression of BS-NOD2 leads to loss of protection from TNBS-colitis) is that the ability of intact NOD2 to protect mice is negated by the fact that the BS-NOD2 transgenic mice express NOD2 that exhibits auto-activation or is hyper-responsive to MDP and thus manifest more persistent TNBS-colitis. To examine this possibility, we inter-bred BS-NOD2 transgenic mice with NOD2-deficient mice to obtain BS-NOD2 transgenic mice on an endogenous NOD2-deficient background. We then isolated cells from these mice to determine their responses to MDP. We found that bone marrow-derived dendritic cells (BMDCs) obtained from NOD2-deficient mice bearing a BS-NOD2 transgene exhibited MDP-induced IL-12p40 and IL-6 responses that were significantly reduced compared to that of BMDCs from WT mice; in addition, baseline (MDP-independent) responses were virtually negative (**Supplementary Figures 2A, B**). Similarly, we found that in Western blot studies NOD2-deficient BMDCs bearing a BS-NOD2 transgene, did not exhibit MDP-independent MAPK and NF-κB responses and exhibited reduced MAP-kinase and absent NF-κB responses upon MDP stimulation (**Supplementary Figure 2C**). Finally, MDP-stimulated cytokine responses of bone marrow-derived macrophages (BMDMs) from NOD2-deficient mice bearing a BS-NOD2 transgene were similar to those of unstimulated cells and MDP-independent responses were not significantly elevated (**Supplementary Figures 2D, E**). These studies indicate that the lack of protection from induction of TNBS-colitis afforded by the BS-NOD2 transgene cannot be explained by NOD2 auto-activation or hyper-responsiveness to MDP stimulation. In addition, these studies provide evidence contrary to the notion that Blau mutations lead to pro-inflammatory hyper-responsiveness elicited by an atypical endogenous NOD2 stimulator other than MDP.

### BS-NOD2 has a dominant-negative effect that interferes with both endogenous NOD2 signaling and cross-regulatory function

**Figure 4:**
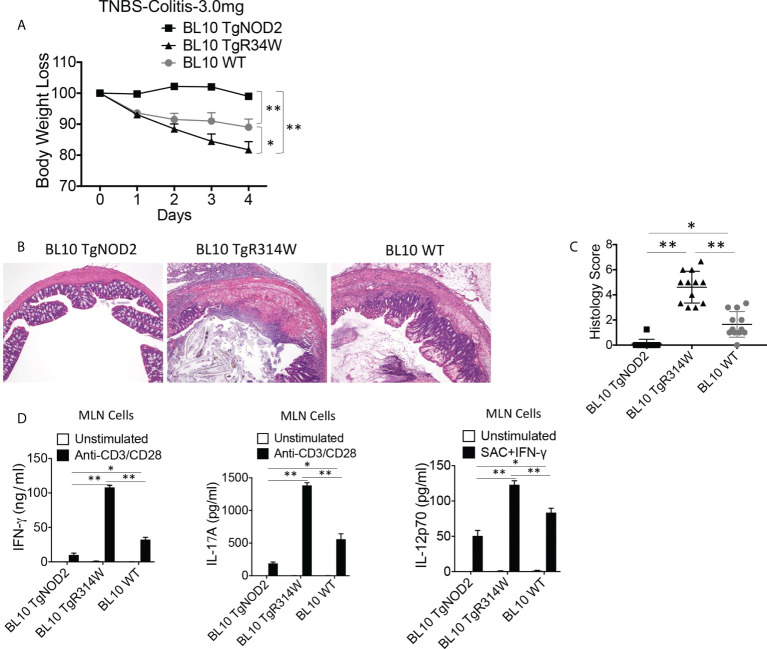
Transgenic BS-NOD2 Has a Dominant-Negative Effect which Interferes with the Cross-Regulatory Function of Endogenous NOD2 in TNBS-Colitis. **(A)** TNBS-colitis associated weight loss of C57BL/10 mice bearing transgenes expressing intact NOD2 (BL10 TgNOD2, n=6) or NOD2-R314W (BL10 TgR314W, n=8) and wildtype (BL10 WT, n=10) mice administered TNBS per rectum (3.0 mg/mouse); mice were harvested for analysis on day 4. Data were analyzed using a two-way ANOVA with Dunnett’s multiple comparisons and are displayed as mean ± SEM; *p<0.05; **p<0.01. **(B)** Representative histology of colons after H&E staining of colonic tissue from mice harvested on day 4. **(C)** Histology scores of mice described in **(A)** plus mice studied in a second identical study involving histologic study only (n=12). **(D)** TCR-induced production of IFN-γ and IL-17A and SAC+IFN-γ-induced IL-12p70 production by extracted MLN cells from mice harvested on day 4; IFN-γ, IL-17 and IL-12p70 production in **(D)** were examined by ELISA. Data in **(C, D)** were analyzed using a two-way ANOVA with Tukey’s multiple comparisons and are displayed as mean ± SEM; *p<0.05; **p<0.01.

The over-expression studies described above did not test the possibility that NOD2 bearing a Blau mutation may exert a dominant-negative effect on the function of co-existing intact NOD2 because the severity of TNBS-colitis induced in the C57BL/10 mice under study was too severe to permit evaluation of greater severity in mice over-expressing Blau-NOD2. To overcome this limitation, we conducted studies with C57BL/10 mice with less severe colitis because the dose of TNBS administered to the mice had been lowered to 3.0 mg/mouse. We found that under these conditions the presence of the BS-NOD2 transgene resulted in more severe colitis than observed in WT mice (**Figures 4A–D**). Similarly, induction of less severe TNBS-colitis in TNBS-colitis-resistant C57BL/6 mice (with in this case administration of 3.75 mg/mouse) led to more severe disease in mice bearing a BS-NOD2 transgene than in either mice bearing a CD-frameshift NOD2 transgene or a WT B6 mice bearing no transgene, whereas mice bearing a CD-frameshift NOD2 and WT B6 mice bearing no transgene exhibited colitis of equal severity (**Supplementary Figures 3A, B**). These *in vivo* data, in showing that the presence of the BS-NOD2 transgene leads to more severe TNBS-colitis than observed in WT mice, suggests that BS-NOD2 over-rides or disturbs the protective effect of endogenous NOD2 as a result of a dominant-negative effect.

**Figure 5:**
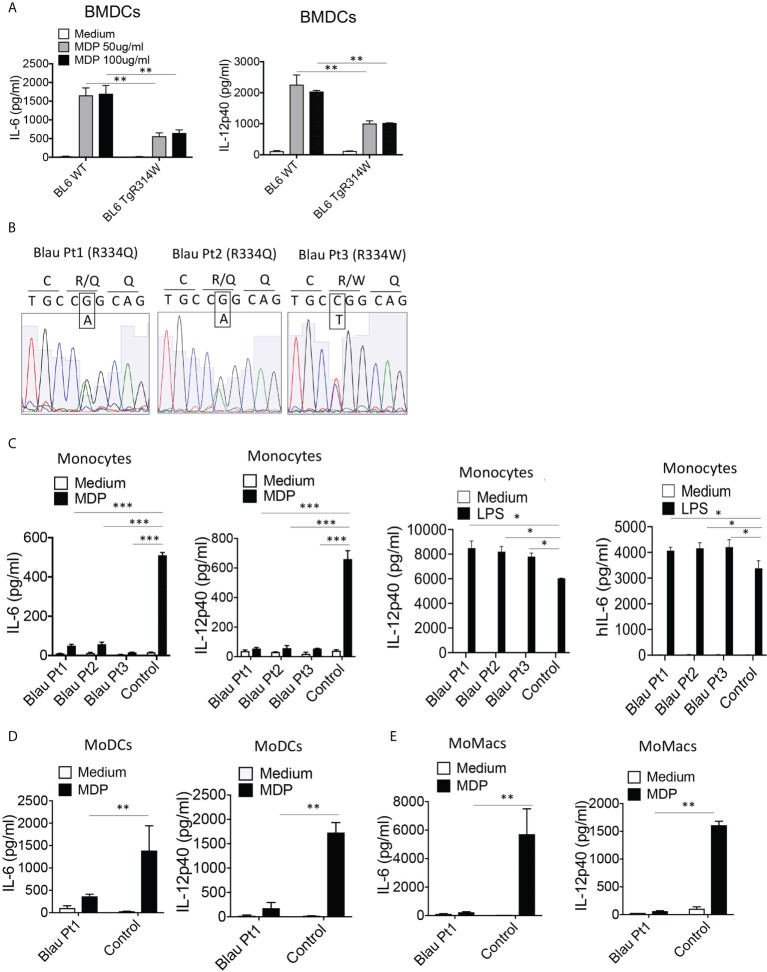
The BS-NOD2 Transgene Exerts a Dominant Negative Effect on Cellular Responses. **(A)** Bone marrow derived dendritic cells (BMDC) from WT (C57BL/6) mice, or C57BL/6 mice expressing a transgene bearing a Blau (R314W) mutation (BL6TgR314W) were stimulated with the indicated doses of MDP for 24 hrs after which the cell culture supernatants were subjected to ELISA analysis of IL-12p40 and IL-6 secretion. **(B)** Total mRNA was extracted from PBMCs of three patients with Blau syndrome (Pt1, Pt2 and Pt3). The RNA samples were reverse transcribed to cDNA and subjected to PCR to amplify human NOD2 sequence. The amplified NOD2 sequence was subjected to sequencing. **(C)** Monocytes isolated from PBMCs of three patients with Blau syndrome (Pt1 and Pt2, carrying R334Q mutation in one allele; Pt3, carrying R334W mutation in one allele of NOD2) and three control individuals were stimulated with MDP (50μg/ml) or LPS (200 ng/ml) for 24 hours after which the cell culture supernatants were subjected to ELISA assay of IL-12p40 and IL-6 secretion. **(D, E)** Monocytes from a Blau patient and a control individual were differentiated into dendritic cells (MoDC, **D**) or macrophages (MoMac, **E**), the cells were then stimulated with MDP (50μg/ml) for 24 hours after which the cell culture supernatants subjected to ELISA assay of IL-6 and IL-12p40 secretion. Data were analyzed using a two-way ANOVA with Tukey’s multiple comparisons and are displayed as mean ± SEM;. *p<0.05; **p<0.01; ***p<0.001.

Further evidence of a BS-NOD2 dominant-negative effect came from perhaps more direct *in vitro* studies that showed that bone marrow-derived dendritic cells (BMDCs) from mice bearing a BS-NOD2 transgene exhibited an approximately 50-60% reduction in cytokine secretion compared to BMDCs from mice bearing WT-NOD2 transgene despite the presence of intact *CARD15* genes encoding WT NOD2 on both endogenous alleles (**Figure 5A**). In addition, MDP stimulation of either peripheral blood monocytes or monocyte-derived dendritic cells (MoDCs) and macrophages (MoMacs) from patients with BS who are heterozygous carriers of the Blau mutation (**Figure 5B**) resulted in far greater reductions in cytokine responses than could be explained by simply having one rather than two alleles encoding NOD2 (**Figures 5C–E**). It should be noted that the decreased MDP-induced IL-6 and IL-12p40 peripheral monocyte responses in **Figure 5C** was accompanied by significantly enhanced LPS-induced IL-6 and IL-12p40 responses (**Figure 5C**, right panels), indicating that diminished cytokine production in Blau patients due to a dominant-negative effect is limited to NOD2-induced cytokine production and that TLR4 (LPS)-induced cytokine responses are increased most likely due to faulty NOD2 cross-regulation (as discussed below).

### Signaling function of NOD2 in cells bearing a knock-in Blau mutation

In a final set of studies that examine the ability of NOD2 with a Blau mutation to cross-regulate TLR responses, we turned to C57BL/6 Blau-Knock-In (Blau-KI) mice originally generated by Dugan et al. that carry a R314Q NOD2 mutation occurring in certain patients with Blau Syndrome (10).

**Figure 6:**
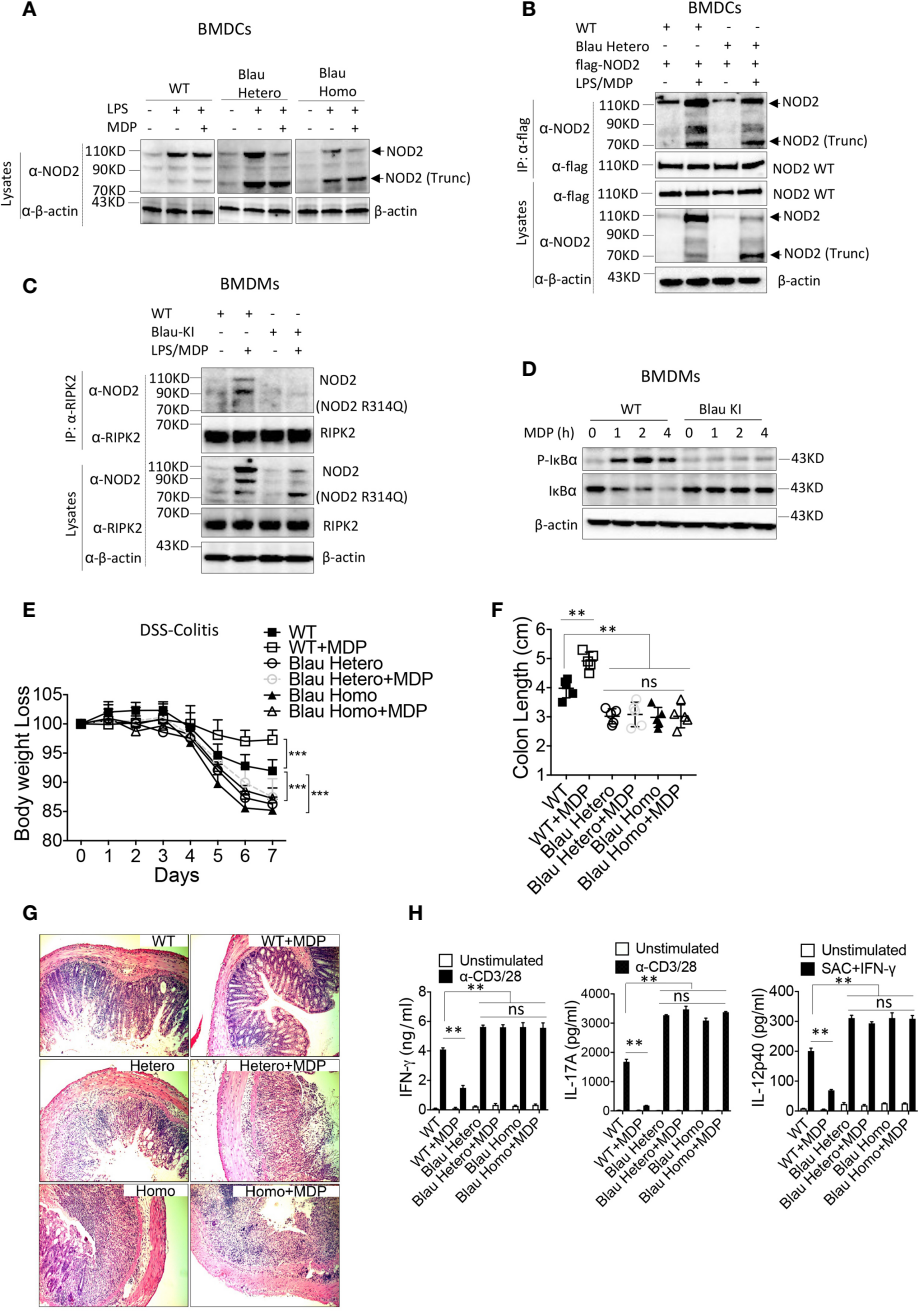
NOD2 bearing a BS mutation expressed on one allele binds to intact NOD2 and exerts a dominant negative effect on intact NOD2 expressed on the other allele of BS-KI heterozygous mice. **(A)** BMDCs from WT mice, heterozygous Blau KI mice or homozygous Blau KI mice were stimulated with LPS (200ng/ml) only or LPS (200ng/ml) plus MDP (50ug/ml) for 6 hours, the cells were lysed, and the cell lysates were then subjected to Western blot for NOD2 detection. **(B)** Bone marrow derived dendritic cells (BMDCs) from WT mice or heterozygous Blau-KI mice were transfected with a plasmid expressing flag-tagged wild type mouse NOD2 as indicated; 48 hours later the cells were primed with LPS (200ng/ml) for 5 hours and then stimulated with MDP (50μg/ml) for 1 hour; the cells were then lysed and the cell lysates were subjected to IP with anti-flag antibody and then Western blot for detection of NOD2 binding forms of endogenous NOD2. **(C)** BMDMs from Blau KI mice or their wildtype littermates were primed with LPS (200ng/ml) for 5 hours and then MDP (50μg/ml) for 1 hours; the cells were then lysed, and the lysates obtained were subjected to IP with anti-RIPK2 antibody and Western blotting for detection of NOD2. **(D)** BMDMs described in **(C)** were incubated with MDP (20μg/ml) for various time periods after which the cells were then lysed and the lysates obtained were subjected to Western blotting for detection of IκBα phosphorylation and degradation. **(E)** DSS-colitis associated weight loss of C57BL/6 heterozygous (n=5) or homozygous (n=5) Blau KI mice and their wild type littermates (n=5) treated with 4% DSS in drinking water; mice were administered muramyl dipeptide (MDP, I00 μg/mouse, IP, n=5) or PBS (n=5) on days 0-5 and were harvested for analysis on day 7. **(F)** Colon length from mice harvested on day 7 after DSS-colitis induction. **(G)** Representative histology of colons after H&E staining of colonic tissue from mice harvested on day 7 after initiation of DSS-colitis induction. **(H)** TCR-induced production of IFN-γ and IL-17A and SAC+IFN-γ-induced IL-12p40 production by extracted MLN cells from mice harvested on day 4 were examined by ELISA. Data in **(A–D)** are representative of three independent experiments. Data in **(E)** were analyzed using a two-way ANOVA with Dunnett’s multiple comparisons and are displayed as mean ± SEM; ***p<0.001. Data in **(F, H)** were analyzed using a two-way ANOVA with Tukey’s multiple comparisons and are displayed as mean ± SEM; **p<0.01; ns: not significant.

In previous studies describing these Blau-KI mice it was shown that whereas stimulated cells from the KI mice produce full-length NOD2 mRNA bearing a Blau mutation (R314Q), they express greatly decreased amounts of full-length NOD2 protein associated with truncated NOD2 protein, most likely the result of increased susceptibility of the full length mutated NOD2 to a proteolytic process that also targets full length un-mutated NOD2 to far lesser extent (10, 23). In Western blot studies we found that BMDCs from homozygous Blau KI mice do express decreased amounts of intact NOD2 and increased amounts of truncated NOD2 particularly when stimulated with LPS and MDP as opposed to LPS alone (**Figure 6A**); the same expression pattern obtains in Western blot studies of heterozygous cells, although in this case the cells express considerably more intact NOD2 presumably resulting (in part) from the non-mutated allele. It should be noted, however, that as shown in immunoprecipitation/immunoblot studies of heterozygous cells, the protein in band designated truncated NOD2 in heterozygous cells binds to intact WT NOD2 and is thereby identified as truncated NOD2 (**Figure 6B**).

In accompanying studies, we determined the ability of BMDMs derived from mice bearing the KI Blau (R314Q) mutation of NOD2 to interact with RIPK2 and support MDP-induced NF-κB activation. Here we found in immunoprecipitation/immunoblot studies that LPS/MDP stimulation of WT BMDMs (derived from littermates of KI mice) induced interaction between NOD2 and RIPK2, whereas such interaction was virtually non-existent in cells from homozygous KI mice expressing BS (R314Q)-NOD2 (with respect to both full length and truncated NOD2) despite the fact that full-length NOD2 was present in lysates probed by anti-NOD2 (**Figure 6C**). Consistent with this finding, MDP-induced IκB phosphorylation was robust in WT BMDCs but was absent in BMDCs from KI mice (**Figure 6D**). These data thus provide additional support for the view that a BS-NOD2 mutation impairs the ability of MDP to induce NOD2 interaction with RIPK2 and thus to cause down-stream NF-κB activation.

### BS-NOD2 Knock-in (Blau-KI) mice display enhanced DSS-colitis and are not protected from colitis by administration of MDP

In further studies we examined the ability of NOD2 present in Blau-KI mice to cross-regulate TLR responses again in the context of gut inflammation. In these studies, we took advantage of the fact that NOD2 cross-regulation can be assessed by intra-peritoneal administration of exogenous MDP so as to cause enhanced NOD2 stimulation and thus inhibition of experimental colitis, such as DSS-colitis or TNBS-colitis (21). Accordingly, we administered MDP (I.P.) to both homozygous and heterozygous Blau-KI undergoing DSS-colitis and then determined the level of colitis in these mouse groups by various criteria as compared to WT mice treated in the same way. We found that both homozygous and heterozygous Blau-KI mice exhibited more severe DSS-colitis than WT littermate control mice as manifested by enhanced body weight loss, colon shortening, microscopic inflammation and enhanced production of pro-inflammatory cytokines by MLN cells (**Figures 6E–H**). In addition, we found that MDP administration ameliorated the severity of colitis in WT mice, but not in either group of Blau-KI mice (**Figures 6E–H**).These data are consistent with the BS-NOD2 over-expression studies described above in suggesting that BS-NOD2 lacks a normal capacity to regulate mucosal inflammation. However, they are subject to the caveat that the NOD2 in the KI mice studied exhibits deficient regulatory function because of mutational effects leading to protein truncation rather than those leading directly to reduced signaling capacity of intact NOD2. This caveat, however, is less applicable to the responses of heterozygous mice since these mice exhibited colitis as severe as that in the homozygous mice yet expressed substantial amounts of unmutated NOD2. In this case, the deficient regulatory function is best attributed to the retained ability of mutated Blau NOD2, despite possible truncation, to interfere with the regulatory function of the unmutated Blau NOD2, i.e., to exert a dominant negative effect.

### The BS-NOD2 mutation causes enhanced TLR responses *in vitro*

**Figure 7:**
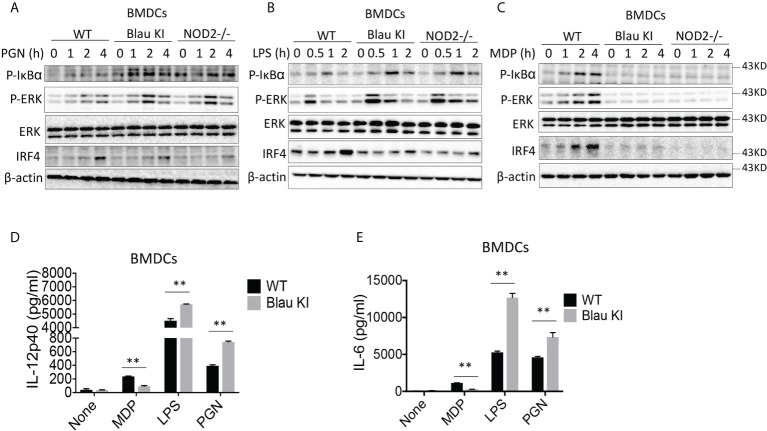
The BS-NOD2 Mutation Causes Loss of NOD2 Cross-Regulation of TLR Responses *in vitro*. **(A–C)** BMDCs obtained from Blau-KI mice, their wildtype littermates and NOD2 KO mice were stimulated with PGN (1μg/ml, **A**), LPS (1μg/ml, **B**) or MDP (50μg/ml, **C**) for various time periods as indicated; the cells were then lysed and the lysates were subjected to Western blotting for detection of phosphorylation of IκBα, ERK, and IRF4. **(D, E)** BMDCs from Blau-KI mice and their wildtype littermates were incubated with LPS (100 ng/ml) or PGN (100 ng/ml) for 24 hours; the culture supernatants were then collected for assay of IL-6 **(D)** and IL-12p40 **(E)** by ELISA. Data in **(A–C)** are representative of three independent experiments. Data were analyzed using a two-way ANOVA with Tukey’s multiple comparisons and are displayed as mean ± SEM; **p<0.01.

Previous studies have shown that NOD2-deficiency and consequent loss of its cross-regulatory ability causes enhanced TLR signaling (13, 14). We therefore investigated the possibility that the inactivating NOD2 Blau mutations lead to enhanced TLR responses. In these studies, we stimulated BMDCs from WT mice, Blau-KI mice and NOD2-KO mice with PGN or LPS and then subjected lysates of the stimulated cells to Western blotting for detection of NF-κB and ERK signaling. We found that the phosphorylation of IκBα and ERK was enhanced in PGN- or LPS-treated Blau-KI cells and NOD2-KO cells as compared with that in WT cells (**Figures 7A, B**). In contrast, both Blau-KI cells and NOD2-KO cells were unresponsive to MDP stimulation (**Figure 7C**). Consistent with these findings, the production of pro-inflammatory cytokines by Blau-KI BMDCs stimulated with PGN or LPS (but not with MDP) were increased compared to that by WT cells (**Figures 7D, E**). These data thus suggest that a Blau-associated mutation or a deficiency of NOD2 is associated with increased TLR activation of NF-κB, MAP kinases and increased production of TLR-induced pro-inflammatory cytokines.

### The BS-NOD2 mutation causes loss of NOD2 cross-regulation of TLR responses *in vivo* at a sterile tissue site

**Figure 8:**
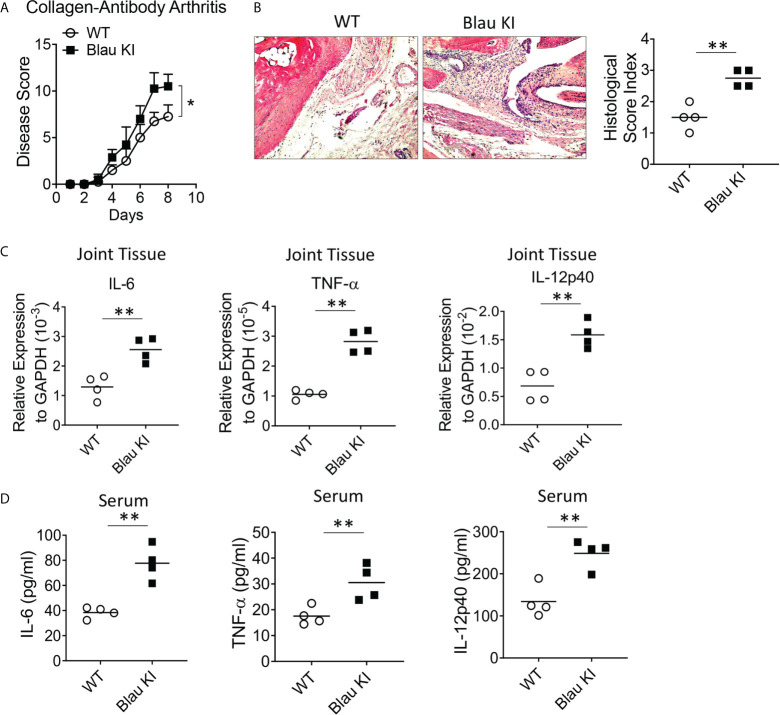
The BS-NOD2 Mutation Causes Loss of NOD2 Cross-Regulation of TLR Responses *in vivo*. **(A, B)** Blau KI mice (homozygous, n=4) and their wildtype littermates (n=4) were administered anti-collagen antibody cocktails (5mg/mouse I.P.) on day 0; the mice were then administered LPS (50ug/mouse, I.P.) on day 3 and were euthanized on day 7. **(B)** Representative histology of joints after H&E staining of tissues from both mice; disease severity was evaluated by arthritis score. **(C)** Joint tissues were harvested for RNA extraction, which were then subjected to reverse transcription and real time RT-PCR for transcription of pro-inflammatory cytokines, IL-6, IL-12p40 and TNF-𝛂. **(D)** Serum samples were collected from mice and concentration of IL-6, IL-12p40 and TNF-𝛂 was examined by ELISA. Data were analyzed using a two-way ANOVA with Tukey’s multiple comparisons; *p<0.05; **p<0.01.

If indeed the Blau mutation leads to enhanced TLR responses due to defective NOD2-mediated cross-regulation *in vitro* as indicated above it should also be associated with enhanced inflammation *in vivo*. That Blau KI mice exhibit more severe DSS-colitis than WT mice as described above is one indication that this is, in fact, the case. To test this possibility in a setting free of an endogenous microflora that could be enhancing inflammation in the absence of NOD2 anti-microbial function (as might be the case in the GI tract) we assessed the severity of anti-collagen antibody-induced arthritis in NOD2 KI mice and WT littermates. Accordingly, we administered anti-collagen antibody cocktails (I.P.) to Blau KI mice and their wildtype littermates and subsequently determined the severity of the induced arthritis and joint histopathology in the two groups. We found that Blau KI mice exhibited significantly greater arthritis by these criteria compared with WT littermates (**Figures 8A, B**). In support of these findings, the transcription of pro-inflammatory cytokines (IL-6, IL-12p40 and TNF-α) was increased in the joint tissue of Blau KI mice compared with that of their wildtype littermates (**Figure 8C**). Moreover, the concentration of these cytokines in Blau KI mice serum was increased as well (**Figure 8D**). These data indicate that the Blau-associated NOD2 mutation confers increased susceptibility to inflammation at a “sterile” site lacking an endogenous microflora that could be influencing the severity of an inflammatory response.

In a second and confirmatory set of studies along these lines we assessed the severity of anti-collagen antibody-induced arthritis in both heterozygous (het) and homozygous (homo) Blau KI mice using the same study protocol as that used in the initial studies described above. In this case we found that both het and homo Blau KI mice exhibit more severe arthritis than WT littermate mice as evaluated by arthritis score (**Supplementary Figure 4A**), ankle histology and inflammation score (**Supplementary Figure 4B**) and joint tissue TNF-α mRNA level (**Supplementary Figure 4C**). The fact that a mutation in the het Blau KI mice causes a level of arthritis almost equivalent to that in the homo KI mice is again indicative of a dominant negative effect.

### The BS-NOD2 mutation is associated with abnormalities of IRF4 expression and function

**Figure 9:**
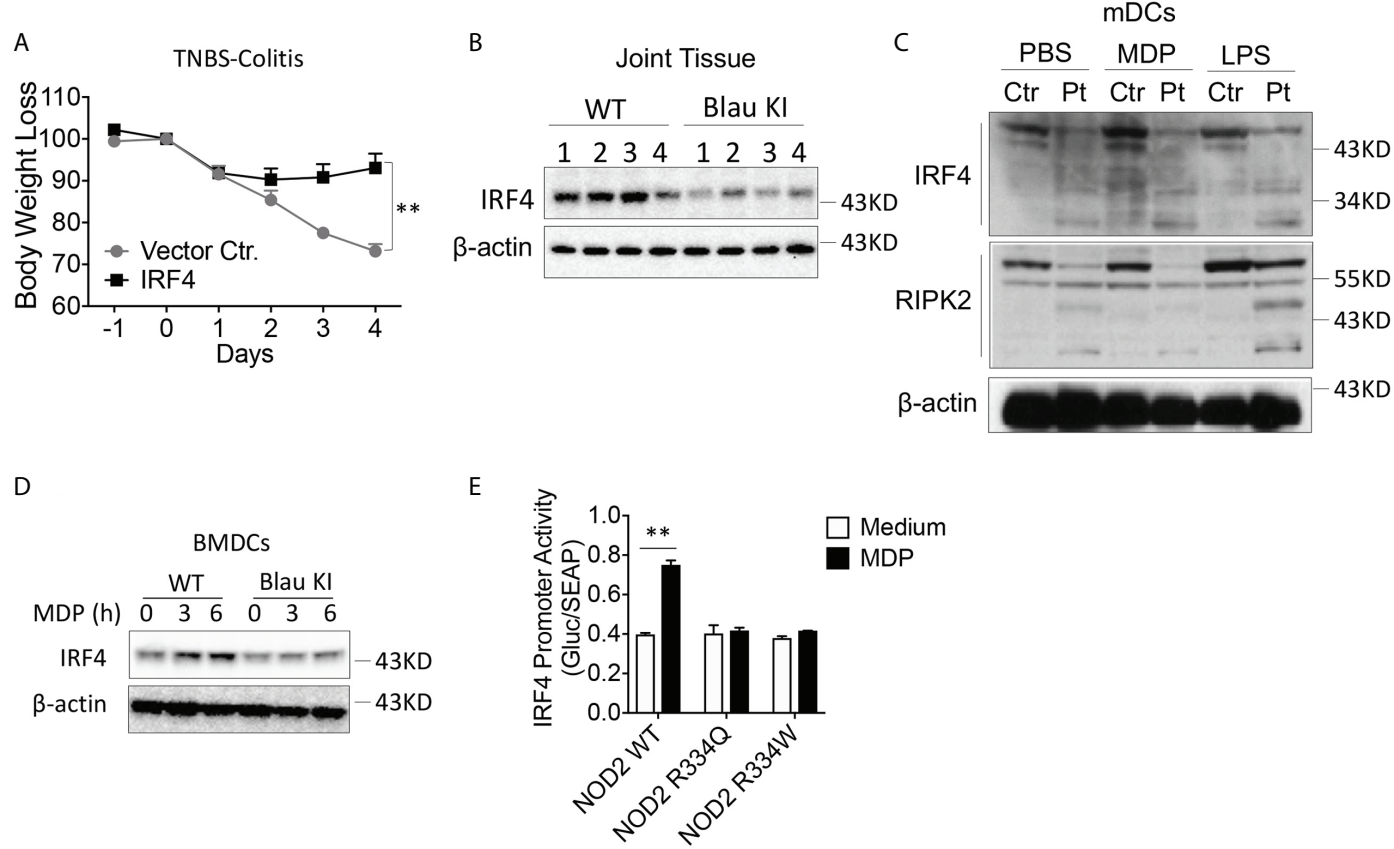
The BS-NOD2 Mutation is Associated with Reduced IRF-4 Expression and Function. **(A)** TNBS-colitis-induced weight loss of C57BL/10 mice administered IRF4 encapsulated in HVJ-E (n=7) or empty vector (Vector Ctr, n=4); TNBS (3.5 mg/mouse) administered per rectum on day 0, IRF4 or control vector administered on day -1, day 0 and day 1; mice harvested for analysis on day 4. **(B)** Blau KI mice (homozygous, n=4) and their wildtype littermates (n=4) were administered anti-collagen antibody cocktails (5mg/mouse by I.P.) on day 0 the mice were then administered LPS (50ug/mouse, by I.P.) on day 3. The mice were euthanized on day 7. Joint tissues were harvested and were homogenized in lysis buffer for protein extraction; the samples obtained were subjected to protein quantification followed by Western blotting for IRF4 detection. 1-4 represents protein samples from individual mouse. **(C)** mDCs from a patient with Blau syndrome or a healthy control individual were treated with or without MDP (50μg/ml) or LPS (100ng/ml) for 6 hours; the cells were then lysed, and the lysates were subjected to Western blotting for detection of IRF4 or RIPK2. **(D)** BMDCs from Blau-KI mice and their wildtype littermates were treated with MDP (50μg/ml, IP) for 3 or 6 hours; the cells were then lysed, and the lysates were subjected to Western blotting for detection of IRF4. **(E)** HEK293T cells were transfected with constructs expressing wildtype NOD2, BS-NOD2 mutations, RIPK2 and IRF4 promoter reporter; 24 hours later, the cells were stimulated with or without MDP (10μg/ml) for 6 hours and were then lysed; finally, the lysates were subjected to luciferase assay for detection of IRF4 promoter activity. Data in **(A)** were analyzed using a two-Two-tailed Student’s t test and are displayed as mean ± SEM; **p<0.01. Data in **(E)** were analyzed using a two-way ANOVA with Tukey’s multiple comparisons and are displayed as mean ± SEM; **p<0.01. Data in **(B–E)** are representative of two independent experiments.

The enhanced TLR-induced phosphorylation of IκBα and ERK in Blau-KI and NOD2-KO cells noted above, was accompanied by reduced TLR-induced expression of IRF4 in these cells as compared to WT cells, particularly in the case of LPS-stimulated cells (**Figures 7A, B**). This observation suggested that the failure to upregulate/activate IRF4 might account for the increased TLR responsiveness observed in cells from Blau-KI mice. Evidence in favor of this possibility comes from previous studies in which we have shown that activated RIPK2 generated by NOD2 signaling interacts with IRF4 and the latter suppresses TLR signaling by acting as a de-ubiquitinating agent that inhibits the ubiquitination of key intermediates necessary for NF-κB activation (16). In addition, we have shown that whereas MDP administration to WT mice inhibits DSS-colitis such administration does not inhibit DSS-colitis in IRF4-deficient mice (21). Finally, in a previous study we have shown that over-expression of IRF4 in C57BL/10 mice using the aforementioned HVJ-E transfection system renders WT mice resistant to the induction of TNBS-colitis (**Figure 9A**) (15).

In the present studies we investigated if NOD2 induction of IRF4 plays a role in the regulation of experimental arthritis induced by anti-collagen antibody described above. We found that the increased level of inflammation in joint tissue observed in mice with a KI Blau mutation exhibit reduced IRF4 levels within inflamed joint tissue as compared with corresponding joint tissue from littermate controls (**Figure 9B**). Evidence that this reduced IRF4 level is directly related to defective NOD2 function came from Western blot studies of monocyte-derived dendritic cells from a patient with BS in which we showed that Blau patient cells express greatly reduced amounts of IRF4 both at baseline and following MDP or, to a lesser extent, following LPS stimulation; in addition, under the same conditions the patient DCs express decreased levels of RIPK2, particularly after MDP stimulation (**Figure 9C**). Similarly, BMDCs from a Blau KI mouse exhibited greatly diminished expression of IRF4 following MDP stimulation (**Figure 9D**). Finally, robust IRF4 promoter activity was found in MDP-stimulated cells expressing wild type NOD2, but not in cells expressing a BS-NOD2 mutation (**Figure 9E**).

### Over-expression of IRF4 restores immune suppression of TLR stimulation in Blau-KI cells

**Figure 10:**
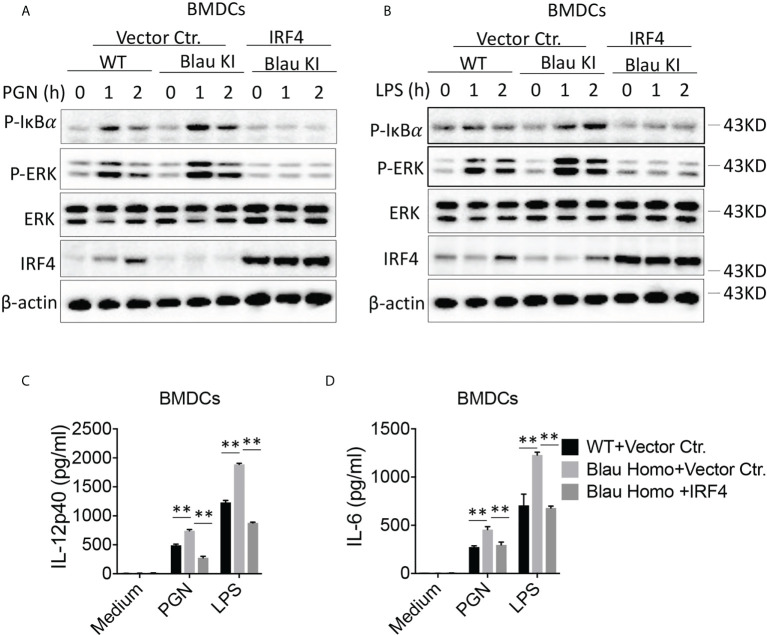
Over-expression of IRF4 Restores Immune Suppression to TLR Stimulation in Blau-KI Cells. **(A, B)** BMDCs from Blau-KI mice and their wildtype littermates were incubated with lentiviral particles expressing mouse IRF4 or empty vector control as indicated for 48 hours; the cells were then stimulated with PGN (1μg/ml, **A**) or LPS (1μg/ml, **B**) for various time periods as indicated; the cells were then lysed and the lysates were subjected to Western blotting for detection of phosphorylation of IκBα, ERK and IRF4. **(C, D)** BMDCs as described in **(A, B)** after transfection with IRF4-expressing constructs were stimulated with LPS (100 ng/ml) or PGN (100 ng/ml) for 24 hours; the culture supernatants were then collected for assay of IL-6 **(C)** and IL-12 **(D)** by ELISA. Data in **(C, D)** were analyzed using a two-way ANOVA with Tukey’s multiple comparisons and are displayed as mean ± SEM; **p<0.01. Data in **(A, B)** are representative of three independent experiments.

In a final series of studies investigating the possibility that the enhanced activation of TLR signaling in Blau-KI cells is due to decreased expression of IRF4 we over-expressed IRF4 in Blau-KI BMDCs using a lentivirus vector expressing an IRF4 construct. Our results showed that infection of Blau-KI BMDCs with an IRF4-expressing lentivirus restored the expression of IRF4 (**Figure 10A**). In addition, we found that after stimulation with PGN or LPS, phosphorylation of IκBα and ERK was increased in empty vector treated Blau-KI cells compared with that in empty vector treated WT cells (**Figures 10A, B**). In contrast, phosphorylation of IκBα and ERK was not enhanced in stimulated Blau-KI cells infected with an IRF4-expressing vector, but instead was reduced below the level in empty vector-treated wildtype cells (**Figures 10A, B**), probably because IRF4 expression and regulatory function in cells treated with IRF4-expressing vector were greater than that in cells treated with empty vector that expressed only endogenous IRF4. A similar result was obtained when the same cells were assessed by their pro-inflammatory cytokine production in that in this case over-expression of IRF4 prevented increased production of cytokines from Blau-KI cells (**Figures 10C, D**). These results are thus again compatible the idea that the enhanced TLR signaling and pro-inflammatory cytokine production in Blau-KI cells is due to reduced IRF4 regulation.

## Discussion

In initial efforts to define the mechanism of inflammation characterizing Blau syndrome we conducted comprehensive *in vitro* studies of the function with NOD2 bearing a range of Blau mutations in its NBD. These studies established that the presence of a Blau mutation compromises the ability of MDP-activated NOD2 to bind to RIPK2 and to induce RIPK2 activation, the latter denoted by phosphorylation and/or K63-polyubiquitination. Not surprisingly these defects ultimately result in a NOD2 molecule that is deficient in its ability to respond to stimulation with MDP with the activation of NF-κB and are consistent with the fact that in this and prior studies cells from Blau patients mount strongly reduced MDP-induced cellular cytokine responses (8–10). These defects also explain the fact, extensively discussed below, that the Blau mutation results in loss of ability to cross-regulate innate immune responses and thus lead to enhanced TLR-mediated pro-inflammatory cytokine responses that contribute to the development of Blau inflammation. An important technical difference between these studies and previous studies was that a plasmid expressing RIPK2 was co-transfected along with a NOD2-expressing plasmid in the luciferase assay whereas in previous studies a RIPK2 plasmid was not co-transfected. However, we found that transfection of plasmids with these BS-NOD2 mutations with and without co-transfection of a RIPK2-expressing plasmid was not associated with increased baseline (MDP-independent) NF-κB activation as compared to that induced by WT NOD2-expressing plasmid

With respect to the defective cross-regulation just mentioned, we and others have shown previously that NOD2 stimulation imposes a negative regulatory effect on concomitant TLR responses (13, 14). Hence, the failure to do so as a result of a loss-of-function LRR-domain (Crohn’s disease-associated) polymorphism is a sufficient reason why such polymorphisms are associated with an increased risk for the development of Crohn’s disease. It was therefore of interest to determine if loss-of-function NBD mutations (Blau syndrome-associated) demonstrated here were also associated with defective TLR response regulation and the inflammations encountered in this syndrome. In accord with prior studies in which NOD2 regulation was assessed, we first determined the ability of over-expressed (intact) WT-NOD2 and/or CD-frameshift-NOD2 versus over-expressed BS-NOD2 to protect mice from the induction of TNBS-colitis. We found that over-expression of intact NOD2, either transiently by the administration of an expression vector encapsulated in a viral envelope or constitutively *via* over-expression of an intact NOD2 by a transgene, leads to very substantial protection from the induction of TNBS-colitis, whereas expression of BS-NOD2 under the same circumstances had a greatly reduced capacity to prevent the development of TNBS-colitis. It was thus established that BS-NOD2 is defective in its ability to exert cross-regulation of pro-inflammatory mucosal responses.

Another observation that emerged from studies of the effect of BS-NOD2 over-expression was that under conditions where the TNBS-colitis induced was relatively mild, over-expression caused more severe colitis than observed in WT mice or in mice that over-express the CD-frameshift-NOD2. Since this was occurring in mice bearing a bi-allelic complement of normal NOD2 genes, this implied that BS-NOD2 but not CD-frameshift NOD2 has a dominant-negative effect that overcomes the endogenous level of protection from experimental colitis normally afforded by NOD2. The fact that the NOD2 with a Blau mutation has a dominant-negative effect was also supported by *in vitro* studies of BS-NOD2 function in the mouse and human bearing Blau mutations where it was observed that responses to MDP were severely impaired in spite of the fact that intact NOD2 was expressed by an endogenous intact NOD2 allele. In these situations, the impairment in MDP responses was more profound than could be explained by the fact that the Blau KI mouse or Blau patients had only a single intact NOD2 allele. This dominant negative effect implies that BS-NOD2 is not completely inert as perhaps is the case with CD-frameshift NOD2, and that MDP elicits some level of NOD2 activation in BS that allow the BS-NOD2 to interact with intact NOD2 in a manner that interferes with the latter’s capacity to exert normal cross-regulatory activity. Evidence for this possibility is inherent in the fact that in NOD2 binding studies it was found that NOD2 with a Blau mutation does bind to intact NOD2, albeit to a lesser extent than intact NOD2 binds to itself. It should be noted, however, that BS-NOD2 may be interfering with normal NOD2 signaling by a mechanism other than binding such as facilitation of an inhibitor of RIPK2 activation.

In a second approach addressing the regulatory function of BS-NOD2 in Blau syndrome and an approach also used in prior studies, we evaluated the ability of NOD2 ligand (MDP) administration to ameliorate DSS-colitis in mice bearing a knock-in mutation in the NBD of NOD2 (KI mice) similar to that found in patients with BS. As discussed above these studies were subject to the caveat that in mice with the mutation on both alleles (homozygous mice) most of the NOD2 protein is present as a truncated molecule probably resulting from increased susceptibility to proteolysis. However, this caveat applies to a lesser extent to mice with a mutation on one allele (heterozygous mice) since in this case one can assess the regulatory function of unmutated NOD2 in the presence of the mutated (mostly truncated NOD2) and thereby evaluate the dominant negative effects of the mutant NOD2 previously demonstrated in the studies of mice bearing NOD2 transgenes. We found that both heterozygous and homozygous mice exhibited more severe DSS-colitis than WT mice and that administration of MDP to these mice did not ameliorate DSS-colitis in such mice although it did so in littermate control animals. A conservative conclusion that can be drawn from this result is that even if the mutated NOD2 did not exhibit cross-regulation because of its truncation rather than an intrinsic loss of function due to the mutation (in the homozygous mice) it was still capable, despite truncation, of inhibiting the regulatory effect of unmutated NOD2 (in the heterozygous mice). As discussed above, this dominant negative effect does not require NOD2 signaling but does require inhibitory interaction with unmutated NOD2. Thus, these studies again showed that whereas BS-NOD cannot itself manifest cross-regulatory function it can inhibit cross-regulatory function of unmutated NOD2.

The above conclusion that NOD2 mutations cause a loss-of-function NOD2 signaling abnormality and an attendant regulatory defect is opposed by the view that the BS-NOD2 mutation is an unalloyed gain-of-function abnormality leading to an excessive pro-inflammatory response. This possibility derives mainly from *in vitro* studies in which it was shown that plasmids expressing NOD2 with mutations found in Blau patients have an increased ability to activate NF-κB reporter constructs in HEK293T cells not stimulated by MDP and thus exhibit auto-activation (4–6). However, this gain-of-function view of the Blau mutational effect was not supported by similar *in vitro* studies reported here in which multiple Blau constructs were shown to lack the ability to activate NF-κB in the absence or presence of MDP stimulation and this signaling defect occurred in cells co-transfected with RIPK2-expressing plasmids which would have enabled such NF-κB signaling. Corroborative evidence that Blau mutations do not directly result in direct gain-of-function defects came from studies of cells from mice bearing a Blau NOD2 transgene in the absence of intact unmutated NOD2 (NOD2 KO mice) or cells from Blau patients. The key finding here was that mouse cells did not exhibit heightened cytokine responses or MAPK/NF-κB responses in the absence of stimulation and exhibited reduced responses following stimulation with MDP; similarly, cells from patients with Blau syndrome did not exhibit increased cytokine production in the absence of MDP stimulation and did exhibit reduced response with MDP stimulation.

The discussion above is strongly reiterative of the point that the Blau mutation is not a direct or immediate cause of a NOD2 gain-of-function abnormality, at least *via* its conventional signaling pathway. However, it may cause the latter indirectly *via* a loss-of-function abnormality affecting negative immune regulation such as that described above. This is exemplified by the fact, already noted above, that mice bearing a BS-NOD2-expressing transgene exhibit more severe TNBS-colitis than WT mice. This view of the Blau abnormality also applies to a recent study in which it was shown that macrophages derived from induced pluripotential stem cells (iPS cells) bearing Blau NOD2 mutations exhibited decreased cytokine responses when exposed to MDP but increased cytokine responses when cultured in the presence of IFN-γ; in addition, cells from one patient with Blau syndrome also exhibited heightened cytokine responses when cultured with IFN-γ (24). These observations were taken as evidence that Blau mutations cause ligand-independent NOD2 hyper-responsiveness and therefore a gain-of-function abnormality not related to MDP-specific NOD2 activation. It is possible, however, that the increased responses to IFN-γ were in reality due to a NOD2 loss-of-function abnormality wherein the mutated NOD2 does not induce activation or upregulation of an inhibitory factor whose absence leads to the increased IFN-γ response.

In studies of the molecular basis of the NOD2 defect underlying BS we showed first that, as discussed above, cells from mice with NOD2 mutations in the NOD2 NBD resulting in signaling *via* RIPK2 and in activation of NF-κB also manifested a defect in the ability to upregulate expression of IRF4, a factor previously identified as necessary for NOD2 cross-regulation (16, 21, 25). These previous studies showed that *in vivo* cross-regulation and protection from colitis is abrogated in IRF4-deficient mice and NOD2 activation facilitates IRF4-mediated de-ubiquitination of key factors necessary for NF-κB activation (16, 21). Finally, these previous studies showed that administration of IRF4 plasmid embedded in an HVJ-E envelope causes IRF4 expression in intestinal cells and inhibition of TNBS-colitis (16). In the present study we showed that whereas administration of MDP to WT mice with anti-collagen arthritis such administration to mice with a KI Blau mutation with DSS-colitis did not; in addition, inflamed joint tissue induced in Blau KI mice by administration of anti-collagen antibody expressed greatly decreased levels of IRF4 as compared to comparable inflamed joint tissue in littermate control mice. Finally, we found that whereas transduction of cells from mice bearing a Blau KI mutation with a lentivirus expressing an empty vector exhibited enhanced IκBα and ERK responses when stimulated *via* TLR2 or TLR4, infection of cells with an IRF4-expressing lentivirus exhibited reduced capacity to activate IκBα and ERK when similarly stimulated. These various studies showing reduced expression of IRF4 in Blau KI mice were corroborated by the observation that stimulated cells from Blau patients also fail to upregulate IRF4. Thus, the combined data provide strong evidence that the inability of mice with a Blau KI mutation to exhibit protection by administration of MDP is attributable to lack of NOD2-mediated IRF4 upregulation.

As already suggested above, the deficiency of cross-regulatory function of NOD2 bearing a Blau mutation is likely to be a contributing factor to the inflammation at non-mucosal body sites in Blau syndrome patients, given the fact that these sites are more or less sterile and therefore not likely to be harboring a robust microflora causing inflammation on its own. This view is compatible with a previous study showing that peptidoglycan-induced uveitis, i.e., non-infectious uveitis, is exacerbated in NOD2-deficient but not NOD1-deficient mice (26) and, more importantly, in the present study showing that anti-collagen antibody-induced arthritis is enhanced in mice bearing a Blau mutation even when the Blau mutation is present on only one allele in heterozygous mice. In humans (and mice) with intact NOD2 the NOD2 regulatory effect could be set in motion by MDP that gains access to the circulation from the gut and thereby induces NOD2 regulatory function at non-mucosal sites. A previous study demonstrating that considerable concentrations of MDP have been detected in the circulation of normal individuals lends credence to this idea (27). However, it is possible that NOD2 exerts regulatory activity in the absence of stimulation by its ligand, MDP. This is evidenced by the fact that BMDCs from BS-KI mice display elevated NF-κB and cytokine responses when stimulated by LPS alone and that lentiviral restoration of IRF4 levels in such cells normalized these increased pro-inflammatory responses. Similarly, in a previous study it was shown that both LPS and poly IC -induced NF-κB/MAPK and cytokine responses were enhanced in the absence of NOD2 most likely because of reduced generation of IRF4 (25). One possible mechanism of this non-MDP-dependent regulatory effect is that NOD2 exerts regulatory function in the absence of RIPK2/NF-κB activation when stimulated by forms of endogenous RNA elicited by TLR stimulation. A study showing that RIPK2-independent NOD2 stimulation induced by ssRNA results in IFN-β production supports this notion (28).

To conclude, studies of the function of Blau-NOD2 conducted here show that the Blau mutation results in NOD2 that does not exert normal canonical signaling *via* RIPK2 and this results in defective activation of NF-κB as well as defective IRF4-dependent NOD2 cross-regulation (i.e., down-regulation) of TLR responses. The latter contributes, at least in part, to enhanced TLR-induced inflammation in various non-mucosal sites. This view of NOD2 dysfunction in Blau syndrome is concisely summarized in the diagram shown in **Supplementary Figure 5**.

## Materials and methods

Complete details on the experimental materials and methods, as well as a description of patients are provided in the supplemental methods.

### Ethics statement

*In vivo* efficacy studies were approved by the Institutional Animal Care and Use Committee of the Rocky Mountain Laboratories (RML). Animal work was conducted adhering to the institution’s guidelines for animal use and followed the guidelines and basic principles in the United States Public Health Service Policy on Humane Care and Use of Laboratory Animals, and the Guide for the Care and Use of Laboratory Animals by certified staff in an Association for Assessment and Accreditation of Laboratory Animal Care (AAALAC) International accredited facility.

### Patients

Blau syndrome patients were recruited for study at the NIH Clinical Center under the NIAID IRB approved protocol 89-I-0153 (http://www.clinicaltrials.gov). A written informed consent has been provided by all patients involved. The R334Q or R334W mutation site was determined by sequencing of patient’s cDNA (**Supplementary Figure 7B**) using primer pairs derived from the published sequence of the NOD2 gene (NP_071445.1). The primers used to amplify NOD2 sequence were as follows: Forward primer: 5’-CTGGAGGAGCTCTTCAGCAC-3’; Reverse primer: 5’-CAGACCTTGGGAAGCTGA GT-3’. The primer used for sequencing was: 5’-CTGGAGGAGCTCTTCAGCAC-3’. Peripheral blood mononuclear cells (PBMCs) of patients and healthy donors were elutriated from the peripheral blood and further purified with magnetic beads obtained from Miltenyi Biotec or human CD4+ T cell enrichment columns obtained from R&D systems to isolate monocytes (CD14+ cells) or helper T cells (CD4+ cells) respectively.

Clinical summary of the three patients with Blau syndrome was shown in supplementary materials.

### Mice

NOD2 deficient mice (NOD2-/- mice) and transgenic mice bearing an intact NOD2 transgene (TgN2 mice) were generated as previously reported (3, 4). Transgenic mice expressing a NOD2-R314W mutation or a NOD2-FS987 mutation were developed at NIH as described below. Blau KI Mice expressing a NOD2 mutation associated with the Blau syndrome (R314Q) were regenerated from frozen sperm originally obtained from KI mice generated by Drs. Michael Davey and James Rosenbaum (5). Wildtype C57BL/6 or C57BL/10 mice were purchased from Jackson Laboratories (Bar Harbor, Maine). Studies of colitis were conducted in mice 8-10 weeks of age. All mice were bred and maintained under pathogen-free conditions at an American Association for the Accreditation of Laboratory Animal Care accredited animal facility at the NIAID and housed in accordance with the procedures outlined in the Guide for the Care and Use of Laboratory Animals under animal study protocol NIAID ASP LCIM13E approved by the NIAID Animal Care and Use Committee.

### Statistics

Data with two groups of samples were analyzed using a two-tailed Students’ t test. Experiments with more than two groups of samples were analyzed using a two-way ANOVA followed by Dunnett’s or Tukey’s *post-hoc* multiple comparisons. Statistical analysis was performed with the GraphPad prism 7. A *p* ≤ 0.05 was regarded as statistically significant. Results are presented as means ± SEM unless otherwise described. Statistical differences between different groups denoted by *: *p* ≤ 0.05; **: *p* ≤ 0.01; ***: *p* ≤ 0.001.

## Data availability statement

The original contributions presented in the study are included in the article/**Supplementary Material**. Further inquiries can be directed to the corresponding author.

## Ethics statement

The studies involving human participants were reviewed and approved by National Institutes of Health Internal Review Board. Written informed consent to participate in this study was provided by the participants’ legal guardian/next of kin. The animal study was reviewed and approved by National Institutes of Health Animal Care and Use Committee.

## Author contributions

LM and GM: Performance of studies and data analysis; writing of MS; AD: Performance of studies and data analysis; IF: Clinical care of patients and planning/analysis of studies; KM-R: Collection of specimens and clinical care; ZY and QX: Performance of studies; AK: Planning and analysis of studies; WS: Planning and analysis of studies; writing of MS. All authors contributed to the article and approved the submitted version.

## Funding

Intramural NIAID Project: ZIA AI000354-37. This work was supported by the Intramural Research Program, National Institutes of Health Clinical Center, National Institute of Allergy and Infectious Diseases, NIH. This study was supported by Intramural National Institutes of Allergy and Infectious Disease Institute Project A1000345-35 and federal funds from the National Cancer Institute, National Institutes of Health, under Contract No. HHSN261200800001E. LM is supported by National Natural Science Foundation of China (32070919) and GM is supported by Natural Science Foundation of China for NOD2 research (81761128012).

## Acknowledgments

The authors thank Drs. Michael Davey and James Rosenbaum for generously providing access to the knock-in mice carrying Blau syndrome associated NOD2 mutation (R314Q). The authors gratefully acknowledge Chuli Yi for her professional technical assistance.

## Conflict of interest

Author KM-R is employed by Leidos Biomedical Research, Inc.

The remaining authors declare that the research was conducted in the absence of any commercial or financial relationships that could be construed as a potential conflict of interest.

## Publisher’s note

All claims expressed in this article are solely those of the authors and do not necessarily represent those of their affiliated organizations, or those of the publisher, the editors and the reviewers. Any product that may be evaluated in this article, or claim that may be made by its manufacturer, is not guaranteed or endorsed by the publisher.

## Author disclaimer

The content of this publication does not necessarily reflect the views or policies of the Department of Health and Human Services, nor does mention of trade names, commercial products, or organizations imply endorsement by the U.S. Government.
