# Supplementary figures and images for: Blau syndrome NOD2 mutations result in loss of NOD2 cross-regulatory function
Source: Front Immunol. 2022 Sep 15;13:988862. doi: 10.3389/fimmu.2022.988862 (PMC9520668; doi:10.3389/fimmu.2022.988862)

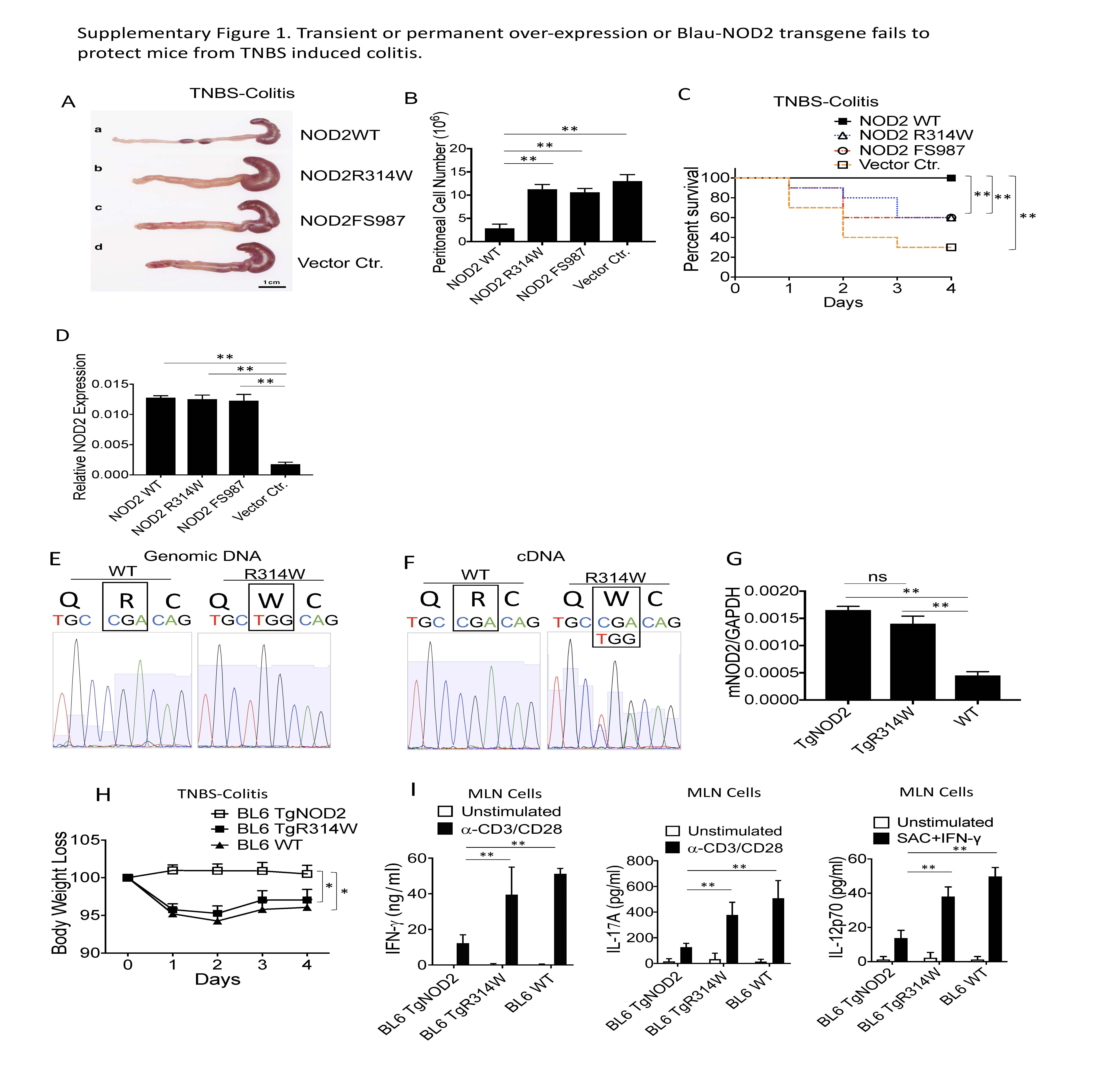

Supplement: Supplementary file 1 [file Image_1.jpeg]

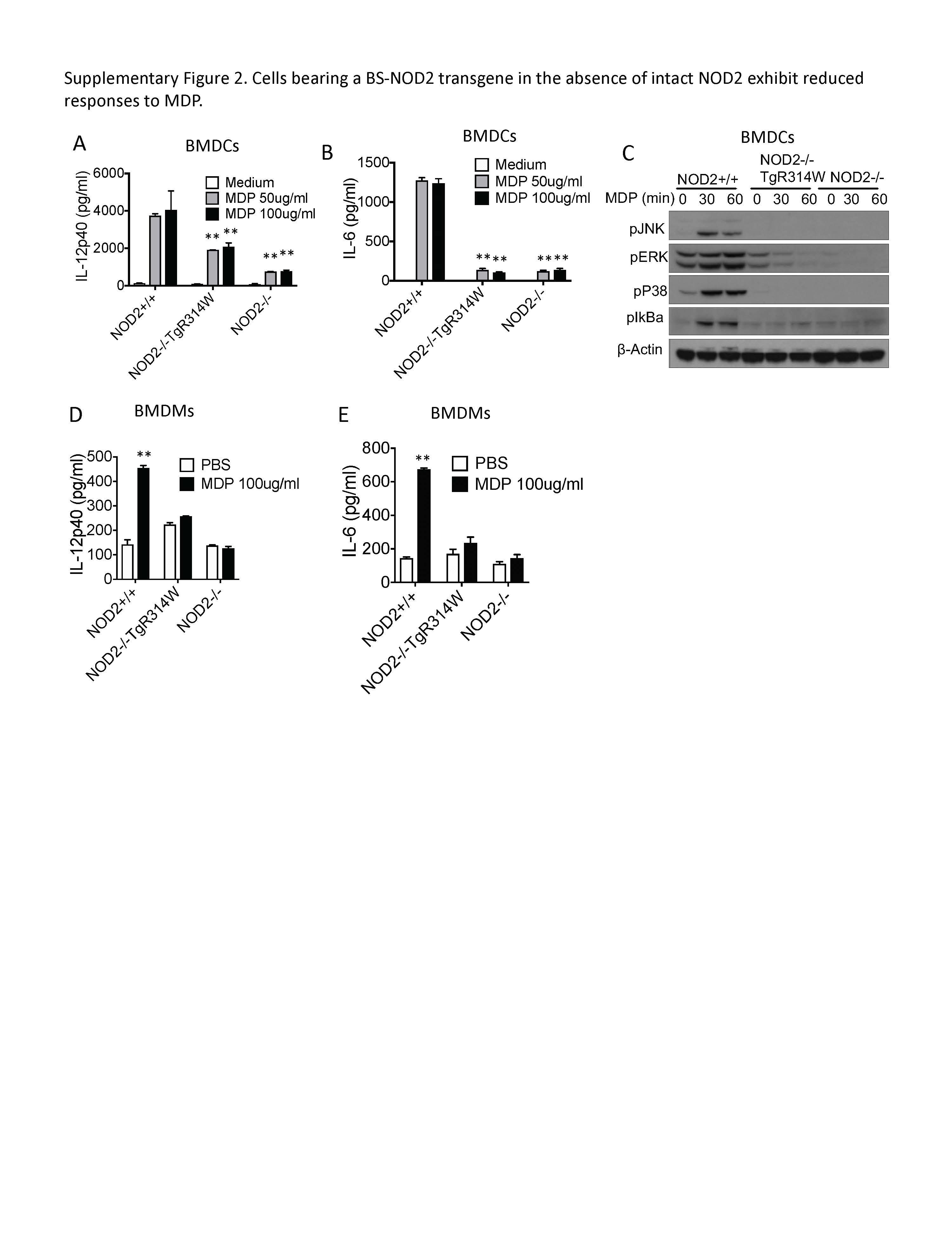

Supplement: Supplementary file 2 [file Image_2.jpeg]

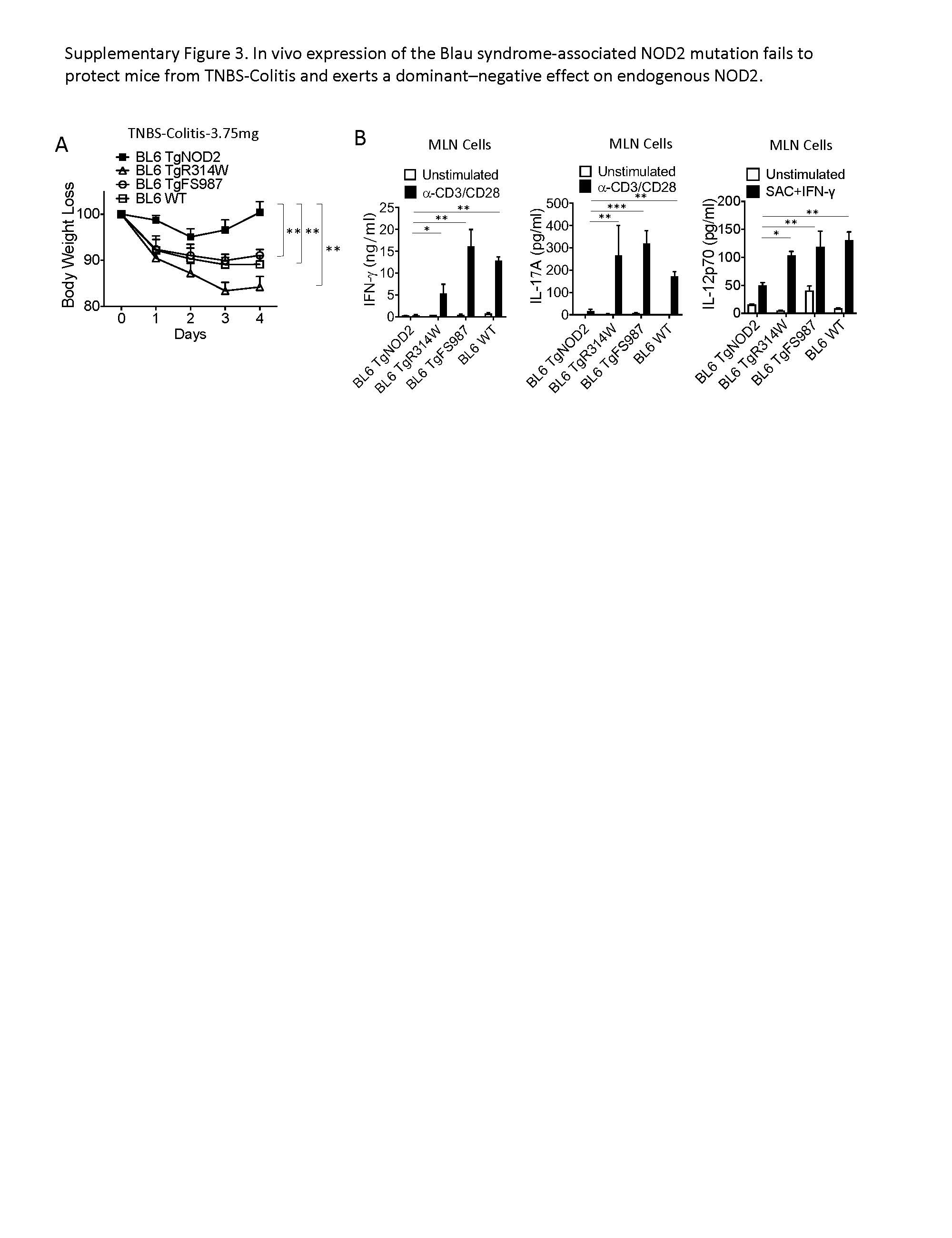

Supplement: Supplementary file 3 [file Image_3.jpeg]

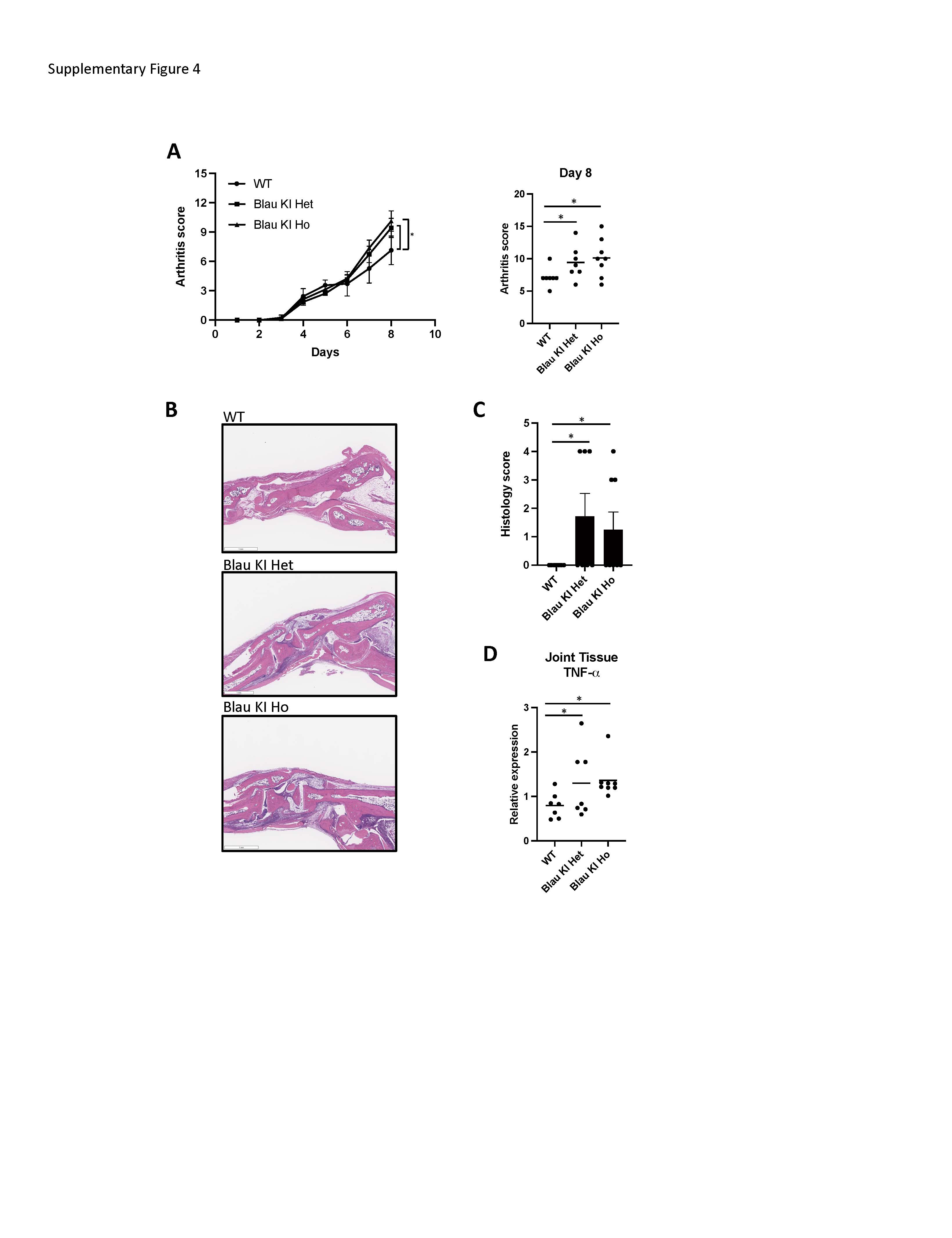

Supplement: Supplementary file 4 [file Image_4.jpeg]

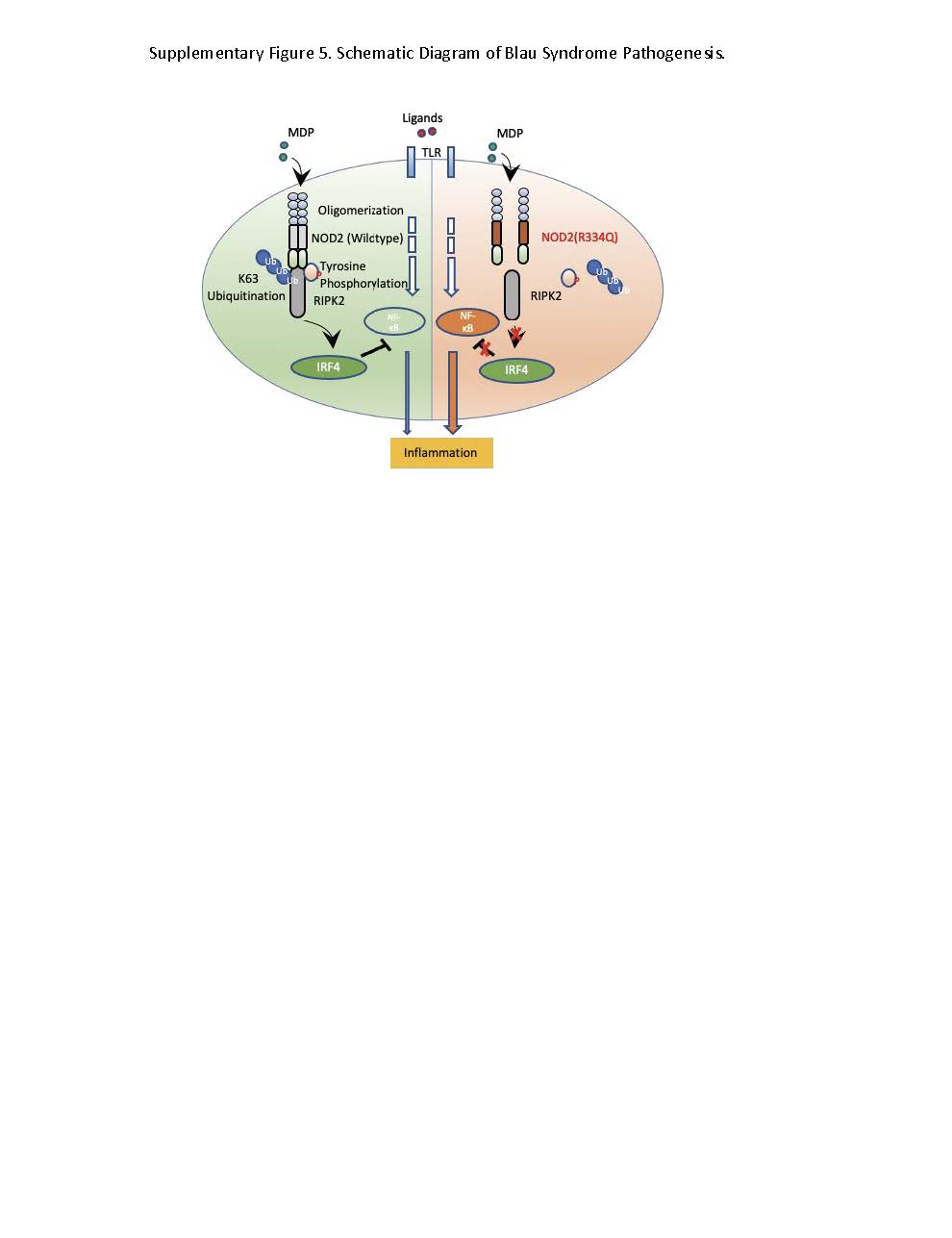

Supplement: Supplementary file 5 [file Image_5.jpeg]
