# Supplementary material for: Blau syndrome NOD2 mutations result in loss of NOD2 cross-regulatory function
Source: Front Immunol. 2022 Sep 15;13:988862. doi: 10.3389/fimmu.2022.988862 (PMC9520668; doi:10.3389/fimmu.2022.988862)
Supplement: Supplementary file 6 [file DataSheet_1.docx]

**Supplementary Materials:**

**Patient information:**

Patient 1(Carrying NOD2 R334Q mutation) is a 42-year-old female who was diagnosed with Juvenile Rheumatoid Arthritis at age 3 years of age with onset arthritis symptoms. She subsequently developed uveitis symptoms at 9 years of age. At age 27, she developed sudden loss of her peripheral vision with slow progression over the years to the point of legal blindness with extensive chorioretinal scarring in the periphery of each eye. She was diagnosed with Blau syndrome after her son at 2 years old, also developed JRA-like symptoms and that prompted her ophthalmologist to obtain genetic testing that revealed the presence of Blau syndrome in both the patient and her son. She is maintained on weekly administration of Humira and methotrexate as well as local treatment of acute uveitis.

Patient 2 (Carrying NOD2 R334Q mutation) is a 19-year-old male, who presented at age 2 with irritability, limping, and the development of trigger finger deformities. He was initially thought to have juvenile rheumatoid arthritis, however, after concomitant uveitis was observed, a diagnosis of Blau syndrome was made. He is maintained on weekly administration of Humira and methotrexate as well as local treatment of acute uveitis.

Patient 3 (Carrying NOD2 R334W mutation) is a 46-year-old male who, at age 12, presented with a rash on his legs and abdomen that disappeared after three months. Subsequently, at age 17, he developed a recurrent erythematous, raised rash after cold exposure and, aat the same time, developed blurred vision which was identified as uveitis. Over the next two decades he was treated intermittently with oral steroids. Blau syndrome was considered when his son developed rash and wrist pain at 9 months of age and this was ultimately confirmed in both father and son by genetic testing. In addition to uveitis, the patient was subsequently noted to have joint pain in the bilateral ankles, knees, and hands. He has been maintained on weekly administration of Humira and methotrexate subcutaneously as well as local treatment of uveitis.

**Reagents and constructs:**

Ultrapure LPS (L3012), peptidoglycan (tlrl-pgns2) and muramyl dipeptide (MDP, A9519) were purchased from Invivogen. Recombinant murine M-CSF (315-02), GM-CSF (315-03), IL-4 (214-14), human M-CSF (300-25), GM-CSF (300-03) and IL-4 (200-04) were purchased from Peprotech. pNTAP-RIPK2 was a gift of Dr. Derek Abbott in Case Western Reserve University School of Medicine. pcDNA3.1D-V5-RIPK2 was provided by Dr. Tomohiro Watanabe(1). pRK5-HA-Ubiquitin-WT (17608), pRK5-HA-Ubiquitin-K63 (K63 only, other lysines mutated to arginines, 17606), pRK5-HA-Ubiquitin-K48 (K48 only, other lysines mutated to arginines, 17605) were purchased from Addgene. pMACS-human NOD2-T7 was generated by subcloning human NOD2 sequence from pUNO1-hNOD2a (Invivogen) to pMACS 4-IRES. II Vector (Miltenyi Biotec). Point mutations of human NOD2 R334Q, R334W, D382E, E383G, C495Y, H496L were introduced using Q5 Site-Directed Mutagenesis Kit (E0554S, New England Biolabs); the primers used are shown in Table 1. pUNO1-mouse NOD2 (puno1-mnod2a) was purchased from InvivoGen. A Flag tag or V5 tag was introduced into the pUNO1-NOD2 plasmid using primers shown in Table 1. NOD2 point mutations were generated using aQ5 Site-Directed Mutagenesis Kit; primers used were shown in Table 1.

Table 1. Primers used in generating mutations of human and mouse NOD2.

| Mutation | Forward Primer | Reverse Primer |
| --- | --- | --- |
| R334Q | AGCAGCTGCAGTGCATGGCCA | GGCAGCTGAATGGGAAGACA |
| R334W | TGGCAGCTGCAGTGCATGGCC | GGCAGCTGAATGGGAAGACA |
| D382E | CGAGTTCAAGTTCAGGTTCAC | GTCAAAGCCATCAAAGGTTAA |
| E383G | ﻿AGTTCAAGTTCAGGTTCACGG | TCGTCAAAGCCATCAAAGGT |
| C495Y | ﻿ACCACCAGGAACTGTTGCTGC | CATTTGGACACCATCCATGA |
| H496L  R314W  R682W  FS987  Flag Insert  V5  Insert | ACCAGGAACTGTTGCTGCAGG  ﻿﻿TGGCAGTTGCAATGCGTGGC  TGGTCGTGTCTGGCCCACAGC  TAGGTTTGGCTTCGAGGGAAC  ﻿GATTACAAGGATGACGACGATAA  -GGCAATGAGATCCAGCTGTTGTGAC  GGTAAGCCTATCCCTAACCCTCTCC  -TCGGTCTCGATTCTACGGCAATGAG  -ATCCAGCTGTTGTGA | TGGCATTTGGACACCATCCA  GCAGCTGAATGGGAAAATGA  GGCACGTGCCTGACGCTGGAG  CAGAATGGCACTGTTCCTGCT  GATGGCCCTCCTACCGGTGATCT  GATGGCCCTCCTACCGGTGATCT |

**Transgene Construction and Generation of Transgenic Mice:**

A pUNO plasmid containing mouse NOD2 cDNA (invivogen) was subjected to site-directed mutagenesis (Stratagene) for generation of R314W (equivalent to human R334W mutation associated with Blau Syndrome) and FS987 (equivalent to human 3020insC mutation associated with Crohn disease) mutations using the following primers: R314Wf, 5’CTCTTCATTTTCCCATTCAGCTGCTGGCAGTTGCAATGCGTGGC CAAACC3’; R314Wr, 5’GGTTTGGCCACGCATTGCAACTGCCAGCAGCTGAA TGGGAAAATGAAGAG3’; FS987f, 5’GCCCTCTTGCAGGCCCTCTAGCAGGAACA GTGCCATTCTGGAG3’; FS987r, 5’CTCCAGAATGGCACTGTTCCTGCTAGAG GGCCTGCAAGAGGGC3’. The pUNO vectors containing the mutated NOD2 were modified for restriction enzyme sites before restriction digestion with EcoRI followed with sub-cloning into pDOI-5 vector at an EcoRI site directly down-stream of a MHC class II promoter (2). Finally, a 7 kb XbaI-EagI fragment of these plasmids containing the MHC-II promoter linked to the mutated NOD2 cDNA was purified and subsequently microinjected into C57BL/6 fertilized oocytes to construct the NOD2-R314W and NOD2-FS987 transgenic mice. Transgenic founder mice were identified by Southern blot analysis of genomic DNA. The founder mice of TgR314W and TgFS987 were then backcrossed with C57BL/10 or NOD2 deficient (N2-/-) mice for 6 generations before experimental application. The genotype of off-spring was determined by PCR screening of murine genomic DNA isolated from tail or ear biopsies. The follow primers were used: NOD2(F): 5’-AGAAGCCCTCCTGCAGGCCC7CA-3’ and Rab-b-globin(R): 5’-CTCAGTGGTATTTGTGAGCCA-3’.

**Real Time RT-PCR analysis of NOD2 expression:**

For real-time RT-PCR analysis of NOD2 expression, total RNA was isolated from spleen cells by RNeasy mini kit (Qiagen, Valencia, CA) and converted to cDNA using the High-Capacity cDNA Archive Kit (Applied Biosystems, Foster City, CA). cDNA (2ug of total RNA per sample) was analyzed in triplicate by TaqMan® PCR using an ABI PRISM 7700 unit (Applied Biosystems) applying 2 sets of probe and primers, with similar results.

Probe A: 5’-(FAM) TGC TTT TTT GCC GCT TTC TAC TTG GCT (TAMRA)-3’,

Forward primer A: 5’-GAG TTC CTG CAC ATT ACC TTC CA-3’,

Reverse primer A: 5’-AGA GGC CAC CGA TGT GTC AG-3’.

Probe B 5'-(FAM) CTGCACAGCTCTGTATTTGCGAGATAACAA (TAMRA)-3'.

Forward primer B 5'GAGATGTCGGAGTGGAACAGC-3';

Reverse primer B 5'-GCGAAGAGCACACTCAACCAG-3';

As an endogenous reference, primers and probe for glyceraldehyde-3-phosphate-dehydrogenase (GAPDH) were used (Applied Biosystems).

**ELISA:**

Cytokine concentrations were determined using BD Biosciences OptEIA ELISA kits for human IL-12p40, murine IL-12p40, IL-6, TNF-𝛂 and IFN-𝛄; eBioScience ELISA kits were used for human IL-6 and murine IL-17A; R&D systems kits were used for murine IL-12p70, human IL-17A and human IFN-𝛄.

**Preparation of Human monocyte derived DCs (MoDCs) and macrophages (MoMacs):**

Monocytes from Blau patients or healthy donors were cultured in 10 cm dishes (1 × 10^6^/ml) in 10 ml of complete medium (RPMI 1640 medium supplemented with 2 mM L-glutamine and 10% fetal calf serum) in the presence of recombinant GM-CSF (20 ng/ml) and IL-4 (20 ng/ml) (Peprotech). After 3 days of culture, half of the medium was exchanged. After 6 days of culture, cells were washed twice and incubated with MDP (Invivogen) or PBS for 24 hours in the absence of GM-CSF and IL-4 before collecting supernatants for ELISA analysis. For generation of MoMacs the same procedures as MoDCs were employed except that M-CSF was used to induce differentiation (40ng/ml).

**Generation of Mouse BMDC and BMDM:**

Bone marrow derived macrophages (BMDM) were prepared as described previously(3). Bone marrow-derived dendritic cells (BMDC) were prepared as follows: total BM cells were cultured in 10 cm cell culture dishes (1 × 10^6^/ml) in 10 ml of complete RPMI medium supplemented with recombinant GM-CSF (20 ng/ml) and recombinant IL-4 (20 ng/ml); after 3 days of culture, half of the medium was exchanged and after 6 days the cells were harvested and selected by anti-CD11c magnetic beads (Miltenyi Biotech). CD11c+ DCs (1 × 10^6^/ml) were incubated with MDP (50 μg/ml) or PBS for 24 hours before collecting supernatants for analysis of cytokine production by ELISA. For western blot experiments, CD11c+ DCs were stimulated with MDP for indicated time periods and then subjected to lysis for gel separation and immunoblotting with indicated antibodies.

**Western blot analysis:**

Cell lysates or beads obtained after IP were mixed with protein loading buffer and then heated at 70℃ for 10 minutes; the mixture components were then separated on NuPAGE Bis-Tris protein gels (Thermofisher scientific) and transferred onto a nitrocellulose membranes (Thermofisher scientific) using a semi-dry transfer system (Bio-Rad); the membranes were then blocked with 5% nonfat milk for 1hr at room temperature after which they were incubated with primary antibodies (as indicated) and washed 3x for 5 minutes each time with TBS containing 0.05% tween-20; they were then incubated with appropriate secondary antibodies conjugated with HRP for 1 hour at room temperature; finally, after washing for 5x for 15 minutes each time with TBS containing 0.05% tween-20, the membranes were incubated in substrate solution (K-12043-D10, Advansta). The immunoreactive bands thus obtained were visualized in a chemiluminescent imager. For detection of MAP kinase and NF-κB activation, murine BMDCs were stimulated (as indicated), after which the cells were lysed in modified lysis buffer (Cell Signaling Technology) in the presence of kinase inhibitors. Total cell lysates were separated on NuPAGE MOPS gel; pJNK (4668s, Cell signaling Technology, Inc.), pP38 (4511s, Cell Signaling Technology, Inc.), pERK (4370s, Cell Signaling Technology, Inc.), pIκB (2859s, Cell Signaling Technology, Inc.) were detected with specific antibodies; the same blots were striped and re-probed with β-Actin antibody (4970s, Cell Signaling Technology, Inc.) as loading controls. For detection of human IRF4 and RIPK2, monocytes or MoDC were either untreated or stimulated with MDP or LPS for 24 hrs, then total cell lysate was prepared and immunoblots were prepared with specific RIPK2 or IRF4 antibodies (RIPK2, Cayman Chemicals, 160785; IRF4, Santa Crutz Biotechnology Inc., sc-6059). For detection of murine IRF4 and RIPK2, mouse colon tissues were homogenized in above buffer after DSS colitis challenge, then total cell lysate was mixed with loading buffer and subjected to immunoblotting with specific RIPK2 or IRF4 antibodies (RIPK2, Cayman Chemicals, 160785; IRF4, Santa Crutz Biotechnology Inc., sc-6059, sc-48338).

**Pull-down Assay and Immunoprecipitation:**

To evaluate the effect of NOD2 mutations on NOD2-RIPK2 interactions, HEK293T cells (2x 10^5^/ml, 1 ml/well) were seeded on 12-well plate in complete medium; then, after overnight incubation at 37℃ they were transfected with 100 ng of plasmids carrying T7-tagged human WT NOD2, or plasmids carrying NOD2 bearing Blau mutations, NTAP-tagged RIPK2 (in the indicated combinations); 24 hours later, the cells were harvested and lysed using a buffer containing 50 mM Tris (pH 7.5), 0.5% NP-40, 50 mM NaCl, 1mM NaVO_4_, 1mM NaF, and protease and phosphatase inhibitor cocktail; the cell lysates were then mixed with agarose beads conjugated with streptavidin (16-126, Sigma-Aldrich) and subjected to pull-down assay. The beads-cell lysate mixtures were incubated on a rotator overnight at 4℃, after which the beads were washed with lysis buffer 3x for 5 minutes each time and subjected to Western blot analysis for detection of NOD2 and RIPK2 using anti-T7 tag antibody (13246S, Cell Signaling Technology, Inc.) and anti-RIPK2 antibody (4142S, Cell Signaling Technology, Inc.), respectively. For detection of RIPK2 tyrosine phosphorylation, anti-tyrosine antibody (9411s, Cell Signaling) was used.

To examine NOD2 intermolecular interaction (homo-oligomerization), constructs expressing Flag-tagged WT or mutated mouse NOD2 (100ng) were co-transfected with a vector expressing V5-tagged WT NOD2 (100 ng) into HEK293T cells (2x 10^5^/ml, 1 ml/well in 12-well plate). 24 hours later the cells were lysed with the buffer described above and the cell lysates were subjected to IP using anti-V5 antibody (R960-25, Thermo Fisher Scientific) and Western blotting for detecti0n of NOD2 using anti-Flag antibody (F1804, Sigma-Aldrich). In a similar assay, either Flag-tagged or His-tagged WT and mutated NOD2 (100 ng for each) were co-transfected into HEK293T cells and 24 hours later, the cells were lysed and the cell lysates were subjected to IP with anti-Flag antibody and Western blotting using anti-Flag antibody or anti-His antibody (sc-8036, Santa Cruz).

In studies examining RIPK2 polyubiquitination, constructs expressing HA-tagged wildtype ubiquitin, K63-only ubiquitin, or K48-only ubiquitin were co-transfected with constructs expressing T7-tagged wild type or mutant NOD2 and V5-tagged RIPK2; then, 24 hours after transfection, the cells were stimulated with MDP (10 μg/ml) for 6 hours after which the cells were harvested and lysed with the buffer described above; the cell lysates were then mixed with agarose conjugated with anti-V5 tag antibody (ab1229, Abcam). For IP assay, the cell lysate-agarose mixtures were incubated on a rotator overnight at 4℃, after which the beads were washed with lysis buffer 3x for 5 minutes each time and subjected to Western blot for detection of RIPK2 ubiquitination using anti-HA antibody (sc-7392, Santa Cruz biotechnology).

To examine NOD2-RIPK2 interaction under physiological conditions, BMDMs (1 X 10^6^/ml, 2 ml/well) from wild type and Blau KI mice were seeded into 6-well plates and incubated overnight on 37℃; the cells were then stimulated with MDP for 24 hours after which they were lysed using the buffer described above; the cell lysates were then subjected to IP by mixing with protein G Dynabeads (10007D, Thermofisher scientific) and anti-RIPK2 antibody (4142S, Cell signaling Technology, Inc.); the resultant mixtures were then incubated on a rotator overnight at 4℃, after which the beads were washed with the lysis buffer 3x5 minutes and subjected to Western blotting using anti-NOD2 antibody (14-5858-81, Thermofisher scientific).

**Induction of Colitis:**

TNBS-colitis was induced by intrarectal administration of TNBS (P2297, Sigma-Aldrich) dissolved in 45% ethanol as previously described (4). After anesthetizing with isofluorane (NDC 10019-360-40, Taxter), the mice were intrarectally administered TNBS solution using a polyurethane catheter positioned 4 cm proximal to the anus; the mice were then held in a vertical position for 30 seconds. Challenged mice were monitored daily for weight loss and then sacrificed on day 4 for histologic assessment of colitis and harvest of MLN cells for assay of inflammatory cytokine production.

For the induction of DSS-colitis, mice were administered drinking water containing 4% DSS for 5 days (day 0-5). The DSS-containing water was replaced by regular water for 2 days (days 6-7). To evaluate the effect of MDP on development of colitis, mice were injected MDP (100 μg/mouse IP) for 5 days (day 0-5). Mice were monitored daily for weight loss and then sacrificed on day 7 for histologic assessment of colitis and harvest of MLN cells for assay of inflammatory cytokine production. Isolated MLN cells were cultured for 48 hours in 48-well plates (10^6^ cells/ml) coated with anti-CD3 (10 µg/ml, BD-Pharmingen) and soluble anti-CD28 (1µg/ml, BD-Pharmingen) for IFN-𝛾 and IL-17 production, and with IFN-𝛾 (1000 units/ml, BD-Pharmingen) and Staphylococcus aureus (1:10,000 Calbiochem, SanDiego, CA) for IL-12p70 production. In some experiments, colon tissues were homogenized and total RNA was extracted with Trizol (invitrogen) and then subjected to RT- PCR for evaluation of expression of indicated cytokines as described before (3).

**Induction of Collagen Antibody Induced Arthritis:**

Blau KI mice (homozygous) and their wildtype littermates were administered anti-collagen antibody cocktails (CIA-MAB-50, ArthritoMab, MDbiosciences, 5mg/mouse by I.P.) on day 0; the mice were subsequently administered LPS (MDLPS derived from E. coli 0111:B4, MDbiosciences, 50ug/mouse, by I.P.) on day 3. Finally, the mice were euthanized on day 7. Disease activity was scored on a 0-4 scale/paw as described previously (5): 0=normal; 1=mild erythema or swelling of the wrist or ankle or erythema and swelling of any severity for 1 digit; 2=more than three inflamed digits or moderate erythema and swelling of the ankle or wrist; 3=severe erythema and swelling inflammation of wrist or ankle; 4=complete erythema and swelling of the wrist and ankle including all digits. For histology analysis, hind ankle joints were dissected, fixed in 10% neutral buffer formalin and were then subjected to decalcification with 10% formic acid-formalin. After embedding in paraffin, the joints were sectioned and subjected to H&E staining. Joint tissues were collected and subjected to RNA extraction, which were then reverse transcribed to cDNA and were subjected to real time RT-PCR for examination of transcription of pro-inflammatory cytokines. Serum was collected for examination of circulating pro-inflammatory cytokines by ELISA. Joint tissues were homogenized in lysis buffer (containing 50 mM Tris (pH 7.5), 0.5% NP-40, 50 mM NaCl, 1mM NaVO_4_, 1mM NaF, and protease and phosphatase inhibitor cocktail) on ice for protein extraction. The samples after homogenation were incubated on ice for 15 minutes and were then subjected to centrifugation at 13,000g for 15 minutes at 4℃. The supernatants were collected for protein quantification and were then subjected to Western blot for IRF4 detection.

**Histological Analysis:**

Mice were euthanized at indicated time points after induction of colitis or arthritis model. The colons or joints were removed and segments were fixed in 10% formalin. After paraffin embedding 5 mm sections were cut and stained with hematoxylin/eosin. Stained sections were examined for evidence of colitis using the following criteria: the presence of lymphocyte infiltration, elongation, and/or distortion of crypts, frank ulceration, and thickening of the bowel wall. The degree of inflammation on microscopic cross-sections of the colon was graded semi-quantitatively from 0 to 4 (0: no evidence of inflammation; 1: low level of lymphocyte infiltration with infiltration seen in a 10% high-power field (hpf), no structural changes observed; 2: moderate lymphocyte infiltration with infiltration seen in 10–25% hpf, crypt elongation, bowel wall thickening which does not extend beyond mucosal layer, no evidence of ulceration; 3: high level of lymphocyte infiltration with infiltration seen in 25–50% hpf, high vascular density, thickening of bowel wall which extends beyond mucosal layer; 4: marked degree of lymphocyte infiltration with infiltration seen in 50% hpf, high vascular density, crypt elongation with distortion, transmural bowel wall-thickening with ulceration) (6). Histological evaluation of arthritis was scored on a 0-3 scale as described previously(7) with minor modifications: 0=normal; 1=mild synovitis; 2=moderate synovitis with pannus formation; 3=severe pannus formation, erosion of bone with fibrin deposition.

Plasmids Expressing intact/mutated NOD2 or IRF4 and their encapsulation for *in vivo* delivery

pUNO plasmids containing an intact/mutated murine NOD2 cDNA mentioned above or murine IRF4 was prepared using an endofree Plasmid Mega kit (Qiagen) and then packaged into a Hemagglutinin Virus Japan-Envelope vector using protamine sulfate (HVJ-E, GenomIdea, Osaka, Japan) according to the manufacturer’s instructions. Mice administered TNBS per rectum were subjected to IP administration of 100 ug of plasmid DNA/mouse encapsulated in 5000 HAU of HVJ-E vector in 100ml of PBS at -1, 0 and +1 day of TNBS intrarectal challenge.

**IRF4 over-expression in BMDCs:**

A lentiviral construct expressing IRF4 was purchased from GeneCopoeia. Viral particles were generated using Lenti-Pac Lentiviral packaging kit (LT001, GeneCopoeia) following manufacturer’s instructions. Briefly, 1.5x10^6^ Lenti-Pac 293Ta cells was seeded into a 10-cm dish in 10 ml of DMEM culture medium with 10% FBS; then, forty-eight hours later, 2.5 µg plasmid expressing IRF4 (pReceiver-Lv181-IRF4, N-flag tag, EX-Mm03333-Lv181) was transfected into the above cells. The cell culture supernatants containing viral particles were harvested 48 hours post-transfection. To concentrate the virus particles, 50 ml original culture supernatant containing virus was mixed with 10 ml Lenti-Pac concentration solution (LT007, GeneGopoeia). The mixture was incubated at 4℃ overnight and then were subjected to centrifugation at 3500g for 25 minutes at 4℃. The virus pellet was resuspend using 10 ml fresh culture medium. To perform transduction, above described BMDCs (10^7^ cells) in differentiation (day 3) were transferred to 10-cm petri dish and incubated with culture medium with concentrated virus particles containing polybrene (8 µg/ml). The cells were incubated at 4℃ for 2 hours and then were transferred to an incubator at 37℃ for 12 hours, after which the virus containing medium was replaced by fresh complete medium without polybrene; the cells were then incubated at 37℃ for 48 hours and then distributed into wells of a 12-well plate at 1x10^6^ cells per well; finally, the cells were stimulated (as indicated) and harvested to examine IRF4 expression using Western blotting.

**Transfection of NOD2 Constructs into BMDCs:**

Constructs expressing Flag-tagged mouse NOD2 were transfected into BMDCs using an Amaxa mouse macrophage nucleofector kit following manufacturer’s instructions. Briefly, 1x10^6^ BMDCs were pelleted and resuspended in nucleofector solution containing 5ug NOD2- expressing construct. The cell containing solution was then transferred into a cuvette and subjected to nucleofection using the Y-001 program. The cell containing solution was then transferred into pre-warmed cell culture medium (2ml) in a 6-well plate. The cells were stimulated and harvested 48 hours post-transfection for use in experiments.

**Luciferase assay for NF-κB or IRF4 promoter activity:**

For NF-κB reporter assay, HEK293T cells (2x10^5^/ml, 1 ml/well) were seeded on 12-well plate in complete medium. After incubating overnight on 37℃, the cells were transfected with constructs expressing a NF-κB luciferase reporter (15ng, E849A, Promega), RIPK2 (65ng), WT-NOD2 (15ng) and BS-NOD2 (15ng); 24 hours later the cells were stimulated with or without MDP (10ng/ml) for 6 hours; finally, the cells were lysed and the lysates were subjected to NF-κB luciferase assay using Dual-Luciferase Reporter assay system (E1910, Promega) following the manufacturer’s instructions. To examine IRF4 promoter activity, HEK293T cells described above were transfected with constructs expressing IRF4 promoter (10ng, HPRM38090, GeneCopoeia), NTAP-RIPK2 (10ng), WT-NOD2 (10ng) and BS-NOD2 (10ng); 24 hours later the cells were stimulated with or without MDP (10ng/ml) for 6 hours; the culture supernatants were then collected for IRF4 protomer assay using Secrete-Pair Dual Luminescence Assay Kit (LF031, GeneCopoeia) following manufacturer’s instructions.

**Statistics:**

Data with two groups of samples were analyzed using a two-tailed Students' t test. Experiments with more than two groups of samples were analyzed using a two-way ANOVA followed by Dunnett’s or Tukey’s post-hoc multiple comparisons. Statistical analysis was performed with the GraphPad prism 7. A *p* ≤ 0.05 was regarded as statistically significant. Results are presented as means ± SEM unless otherwise described. Statistical differences between different groups denoted by *: *p* ≤ 0.05; **: *p* ≤ 0.01; ***: *p* ≤ 0.001.

**Supplementary References:**

1. Watanabe T, Asano N, Meng G, Yamashita K, Arai Y, Sakurai T, et al. NOD2 downregulates colonic inflammation by IRF4-mediated inhibition of K63-linked polyubiquitination of RICK and TRAF6. *Mucosal Immunol.* 2014;7(6):1312-25.

2. Kouskoff V, Fehling HJ, Lemeur M, Benoist C, and Mathis D. A vector driving the expression of foreign cDNAs in the MHC class II-positive cells of transgenic mice. *J Immunol Methods.* 1993;166(2):287-91.

3. Meng G, Zhang F, Fuss I, Kitani A, and Strober W. A mutation in the Nlrp3 gene causing inflammasome hyperactivation potentiates Th17 cell-dominant immune responses. *Immunity.* 2009;30(6):860-74.

4. Yang Z, Fuss IJ, Watanabe T, Asano N, Davey MP, Rosenbaum JT, et al. NOD2 transgenic mice exhibit enhanced MDP-mediated down-regulation of TLR2 responses and resistance to colitis induction. *Gastroenterology.* 2007;133(5):1510-21.

5. Hutamekalin P, Saito T, Yamaki K, Mizutani N, Brand DD, Waritani T, et al. Collagen antibody-induced arthritis in mice: development of a new arthritogenic 5-clone cocktail of monoclonal anti-type II collagen antibodies. *J Immunol Methods.* 2009;343(1):49-55.

6. Scheiffele F, and Fuss IJ. Induction of TNBS colitis in mice. *Curr Protoc Immunol.* 2002;Chapter 15:Unit 15 9.

7. Nandakumar KS, Svensson L, and Holmdahl R. Collagen type II-specific monoclonal antibody-induced arthritis in mice: description of the disease and the influence of age, sex, and genes. *Am J Pathol.* 2003;163(5):1827-37.

**Supplementary Legends:**

**Supplementary Figure 1. Transient or permanent over-expression or Blau-NOD2 transgene fails to protect mice from TNBS induced colitis.**

(A) Colon morphology of C57BL/10 mice subjected to TNBS-colitis and harvested on day 4 after initiation of colitis induction; as described in Figure 3A, shortened and thickened colons reflect inflammation. (B) Peritonitis of mice with TNBS-colitis administered indicated plasmids via encapsulated in HVJ-E as described in Figure 3A, indicating different levels of peritoneal inflammation. (C) Survival rates of mice with TNBS-colitis transfected with indicated NOD2 plasmids as described in Figure 3A. (D) RT-PCR analysis of intact or mutated NOD2 expression from spleen cells isolated from mice transfected with indicated plasmids encapsulated in HVJ-E, indicating comparable expression levels of the different NOD2 plasmids. (E-F) Sequence analysis of genomic DNA (E) or cDNA reverse transcribed from total mRNA (F) derived from NOD2-R314W transgenic mice, indicating the successful integration of NOD2 bearing a R314W mutation. (G) RT-PCR analysis of RNA extracted from spleens of mice with transgenes bearing intact NOD2 or NOD2 with a R314W mutation, indicating comparable expression levels of the different NOD2 transgenes. (H) Weight loss of transgenic C57Bl/6 mice per rectal administration of TNBS (3.5mg/mouse) for colitis induction; mice were harvested on day 4. (I-J) IFN-𝛄 and IL-17 production by TCR stimulation of MLN cells of mice harvested on day 4 after TNBS challenge as described in (H). (K) IL-12p70 production by SAC+IFN-𝛄 stimulated MLN cells of indicated mice harvested on day 4 after TNBS challenge as described in (H). Data in C were analyzed by Log-rank (Mantel-Cox) test. Data in B, D and G were analyzed using a one-way ANOVA with Dunnett’s multiple comparisons and are displayed as mean ± SEM; ***p*<0.01; Data in H were analyzed using a two-way ANOVA with Dunnett’s multiple comparisons and are displayed as mean ± SEM; Data in I-K were analyzed using a two-way ANOVA with Tukey’s multiple comparisons and are displayed as mean ± SEM; ***p*<0.01.

**Supplementary Figure 2. Cells bearing a BS-NOD2 transgene in the absence of intact NOD2 exhibit reduced responses to MDP.**

(A-B) Bone marrow derived dendritic cells (BMDCs) from WT (NOD2+/+) mice, NOD2-deficient (NOD2-/-) mice, or endogenous NOD2-deficient mice expressing a transgene (R314W) mutation (NOD2-/-TgR314W) were stimulated with the indicated doses of MDP for 24 hours and then evaluated for IL-12p40 (A) and IL-6 (B) secretion by ELISA. (C) BMDCs described in (A) were stimulated with MDP (100μg/ml) for the indicated time followed with immunoblotting for activation of MAP kinases and NF-κB; β-actin blotting served as loading control. (D-E) Bone marrow derived macrophages (BMDM) from WT (NOD2+/+) mice, NOD2 deficient (NOD2-/-) mice, or endogenous NOD2-deficient mice bearing transgene expressing a R314W mutation (NOD2-/-TgR314W) were incubated with the indicated doses of MDP for 24 hours after whichIL-12p40 (D) and IL-6 (E) secretion into culture supernatant was analyzed by ELISA. Data were analyzed using a two-way ANOVA with Dunnett’s multiple comparisons and are displayed as mean ± SD; ***p*<0.01. All data are representative of three independent experiments.

**Supplementary Figure 3. *In vivo* expression of the Blau syndrome-associated NOD2 mutation fails to protect mice from TNBS-Colitis and exerts a dominant–negative effect on endogenous NOD2.**

(A) Weight loss of C57BL/6 transgenic mice with transgenes bearing intact NOD2 (TgNOD2), NOD2 with Blau syndrome associated mutation (TgR314W) or NOD2 with frameshift mutation (TgFS987) transgenic mice and C57BL/6 wildtype mice upon rectal administration of TNBS (3.75 mg/mouse) for colitis induction; mice were harvested for analysis on day 4. (B) Responses of MLN cells extracted from the mice at harvest following stimulation with TCR or SAC+IFN-𝛄 to assess IFN-𝛄, IL-17A and IL-12p70 secretion into culture supernatants (examined by ELISA). Data were analyzed using a two-way ANOVA with Dunnett’s multiple comparisons and are displayed as mean ± SEM; **p*<0.05; ***p*<0.01; ****p*<0.001.

**Supplementary Figure 4. Anti-Collagen Antibody Arthritis in BS-NOD2 KI Heterozygous and Homozygous Mice.**

(A) Blau KI mice (Heterozygous, n=7; homozygous, n=8) and their wildtype littermates (n=7) were administered anti-collagen antibody cocktails (4,5 mg/mouse I.P.) on day 0; the mice were then administered LPS (50ug/mouse, I.P.) on day 3 and were euthanized on day 8. The disease severity was evaluated by arthritis score. (B)Representative histology of H&E stained joint tissues from mice after induction of arthritis model in A. (C) Histology scores of three groups of mice after induction of arthritis model in A. (D) Joint tissues were harvested from mice in A for RNA extraction, which were then subjected to RT-PCR for assessment of TNF-α mRNA. Data were analyzed using a two-way ANOVA with Tukey’s multiple comparisons; **p*<0.05; ***p*<0.01.

**Supplementary Figure 5: Schematic Diagram of Blau Syndrome Pathogenesis.**

Blau NOD2 mutations cause defects in canonical signaling involving RIPK2 and downstream NF-kB activation; this results in defective IRF4 upregulation and loss of cross-regulation of innate responses. Schematic diagram below shows intact NOD2 stimulation and signaling leading to a moderate (regulated) inflammatory response on the left (green) side and lack of such signaling leading to excessive (poorly regulated) inflammatory response on the right (orange) side.
